# Global, regional, and national burden of musculoskeletal disorders, 1990–2021: an analysis of the global burden of disease study 2021 and forecast to 2035

**DOI:** 10.3389/fpubh.2025.1562701

**Published:** 2025-08-01

**Authors:** Meizhi Liu, Jian Rong, Xiangzhen An, Yulei Li, Yan Min, Guomeng Yuan, Yan Yang, Mengjie Li

**Affiliations:** ^1^Department of Rheumatology, Hebei Provincial People’s Hospital, Shijiazhuang, China; ^2^Department of Scientific Research, The Second Affiliated Hospital of Anhui Medical University, Hefei, China; ^3^Department of Rheumatology, Bai Qiu’en International Peace Hospital, Shijiazhuang, China; ^4^Department of Clinical Pharmacy, Shenyang Pharmaceutical University, Shenyang, China; ^5^Department of Psychology, The First Affiliated Hospital of the Chinese People’s Liberation Army Air Force University of Military Medicine, Shaanxi, China; ^6^Department of Information, North China Medical and Healthcare Group Huayao Hospital, Shijiazhuang, China

**Keywords:** musculoskeletal disorders, epidemiology, global burden of disease, frontier analysis, Bayesian age-period-cohort model

## Abstract

**Objectives:**

This study aimed to assess the global, regional, and national burdens of musculoskeletal disorders (MSDs) since 1990. It also projected trends up to 2035 and explored potential improvements through frontier analysis.

**Methods:**

Using data from the Global Burden of Disease 2021 study, MSDs in 204 countries and territories were analyzed. Age-standardized rates (ASRs) for prevalence (ASPR), incidence (ASIR), DALYs (ASDR), and mortality (ASMR) were calculated. Trends were evaluated through estimated annual percentage changes (EAPC). The Bayesian age-period-cohort (BAPC) model was employed for projections to 2035, and frontier analysis was used to assess the potential for reducing MSD burdens.

**Results:**

In 2021, 1.686 billion MSDs prevalent cases were recorded globally, representing a 95% increase since 1990. Although total cases and DALYs have increased, ASIR and ASMR showed declining trends, with global MSD-related mortality decreasing by 0.265% annually. By 2035, the number of MSD cases is projected to rise to 2.161 billion, along with corresponding increases in DALYs and mortality, although ASRs are expected to continue declining. Frontier analysis revealed significant gaps between current burdens and achievable benchmarks, particularly in high-SDI regions, while some low-SDI regions demonstrated effective management despite limited resources. A U-shaped relationship between SDI and MSDs burdens was observed, with middle-SDI regions generally performing better.

**Conclusion:**

The global burden of MSDs is projected to rise in absolute case numbers, underscoring the necessity for strategically targeted interventions to manage their impact effectively. Frontier analysis illuminates potential improvements, particularly in high-SDI countries, while projections indicate that enhanced access to healthcare and better resource distribution could alleviate the global MSDs burden. Addressing disparities and implementing tailored interventions are crucial for reducing MSDs-related disability and mortality.

## Introduction

Musculoskeletal disorders (MSDs), including rheumatoid arthritis (RA), osteoarthritis (OA), low back pain (LBP), neck pain (NP), and gout, represent a group of conditions that impose substantial challenges on global healthcare systems (1). These disorders are characterized by symptoms such as pain, limited mobility, diminished flexibility, overall functional impairment, and decreased work capacity (2). MSDs are associated with a high incidence and disability rate (3). According to the Global Burden of Disease (GBD) study, MSDs account for 6.7% of global disability-adjusted life years (DALYs), with this figure rising to 21.3% in high-income countries (4). In 2017, non-communicable diseases were responsible for 80% of disabilities (5), with specific conditions like neck pain (18.4%), osteoarthritis (19.3%), and low back pain (36.8%) being highly prevalent worldwide, thus placing a significant strain on public health (2, 6). By 2020, MSDs had become the second leading cause of non-fatal disability, affecting over 1.63 billion people globally (4). These diseases significantly impair the physical, psychological, and functional status of individuals, posing a considerable threat to healthy aging (7, 8). They contribute not only to a substantial number of years lived with disability but also to premature mortality, particularly in cases of rheumatoid arthritis, osteoarthritis, and low back pain (5, 6, 9). The burden of MSDs varies significantly across different countries and regions (10).

The GBD study provides essential data for comprehensively assessing the global disease burden, facilitating the development of targeted interventions, optimizing resource allocation, and understanding major public health challenges (11). MSDs severely restrict individuals’ activities and social participation (12), limit their work and retirement capabilities, and increase the demand for social and economic support (13). Additionally, factors such as population aging, sedentary lifestyles, obesity, and injuries are expected to escalate the disability burden attributable to MSDs (5). Despite several GBD publications documenting the burden of specific MSDs prior to 2021 (14, 15), a comprehensive analysis of the overall burden and trends of MSDs was lacking, highlighting a significant research gap that needed further exploration. Understanding the overall burden and trends of these diseases is crucial for formulating targeted interventions and optimizing resource allocation.

This report systematically analyzes the global, regional, and national burden of MSDs from 1990 to 2021, utilizing data from the GBD 2021 database. For the first time, it applies frontier analysis to explore the relationship between the MSDs burden and the level of social and demographic development across different countries, and projects the prevalence trends up to 2035.

## Methods

### Data acquisition

This study utilizes data from the GBD 2021 dataset, an exhaustive repository assessing the global and regional consequences of 371 health conditions, injuries, and impairments, along with 88 risk factors, across 204 countries and territories spanning from 1990 to 2021 (16). Encompassing the expertise of thousands of international collaborators, the GBD 2021 offers detailed insights into the incidence, prevalence, mortality, and DALYs associated with diverse health conditions. For this analysis, data specifically pertaining to MSDs were extracted using the Global Health Data Exchange (GHDx) tool (https://vizhub.healthdata.org/gbd-results/), with a focus on variables such as age, sex, geographic location, and risk-attributed DALYs. Additionally, sociodemographic data, encapsulated by the Sociodemographic Index (SDI) (https://ghdx.healthdata.org/gbd-2021), were collected to examine the impact of economic and social factors on the burden of MSDs. The SDI, a composite indicator derived from income, educational attainment, and fertility rates, classifies countries into five developmental categories—low, low-middle, middle, high-middle, and high. This classification facilitates a deeper understanding of health disparities across various settings.

### Data sources and disease model

The GBD 2021 study employed a diverse array of data sources to estimate mortality and disease burden from MSDs. These sources included vital registration systems, disease surveillance databases, health records, and population surveys. Rigorous criteria were applied to eliminate outlier data points that deviated significantly from global or regional patterns, were inconsistent with expected age or temporal trends, or exhibited discrepancies when compared to data from similar settings. To estimate mortality associated with MSDs, the Cause of Death Ensemble Model (CODEm) was utilized. CODEm synthesizes data from multiple sources and employs Bayesian inference techniques to select the most suitable models, taking into account variations by region, age, and sex. Covariates were chosen based on their demonstrated relationship with MSDs mortality, including average body mass index (BMI), alcohol consumption rates, smoking prevalence, healthcare access quality, and bone mineral density levels. The influence of each covariate (whether positive or negative) was determined based on the robustness of the evidence supporting their association with mortality rates. The prevalence and overall disease burden of MSDs were estimated using the DisMod-MR 2·1 model, which adjusts for regional, temporal, and demographic differences in the data, incorporating age-specific priors to improve the model’s precision. Further details on the modeling techniques are available in the GBD 2021 methods appendices (https://www.healthdata.org/gbd/methods-appendices-2021).

### Estimation of disease burden

In 2021, a comprehensive analysis was conducted to assess the burden of MSDs, focusing on their incidence, prevalence, DALYs, and mortality rates. Additionally, this study examined the influence of demographic and socioeconomic factors, including age, gender, and the SDI, on the distribution of MSDs. Four principal metrics were employed to measure this burden: the age-standardized incidence rate (ASIR), age-standardized prevalence rate (ASPR), age-standardized DALY rate (ASDR), and age-standardized mortality rate (ASMR). To delineate long-term trends, the EAPC in these rates was computed for the period from 1990 to 2021.

The EAPC is a standard method for evaluating temporal trends in disease burden. It is calculated using a log-linear regression model on the natural logarithm of the ASRs, expressed as ln (ASRs) = a + bx + e, where ln (ASRs) denotes the natural logarithm of the ASRs, x represents the calendar year, a is the intercept, b is the slope, and e is the error term. The EAPC is then determined using the formula: EAPC = [exp(b) – 1] × 100, with a 95% confidence interval (CI) estimated from the regression. ^16^ Trends are classified as decreasing if the upper limit of the CI is less than zero, and as increasing if the lower limit exceeds zero. This methodology provides a comprehensive overview of the evolving burden of MSDs, including variations across different age groups and socioeconomic contexts, thus informing targeted public health strategies to address the global and regional impacts of MSDs.

### Decomposition analysis

To investigate the relationship between MSDs burden and socioeconomic development, as indicated by the SDI, we utilized frontier analysis to model the ASRs. Unlike conventional regression techniques, frontier analysis accommodates the non-linear association between the SDI and disease burden and identifies multifaceted contributing factors. This approach delineates the theoretical minimum ASRs that each country or region could achieve at its current level of development, establishing a benchmark for optimal performance. It further quantifies the disparity between the actual burden and the theoretical minimum, thereby identifying areas needing improvement (17). To achieve this, we integrated locally weighted regression with local polynomial regression, applying a smoothing span of 0.3 to create a smooth, non-linear boundary curve between the SDI and ASRs. To ensure methodological robustness, 1,000 bootstrap samples were conducted, and the mean ASRs for each SDI value were calculated. By measuring the absolute distance between the actual ASRs of each country in 2021 and the boundary curve, we evaluated the potential for reducing the MSDs burden across various countries.

### Predictive analysis

To inform public health policy and resource allocation, we utilized the BAPC analysis model. This model, implemented via the INLA and BAPC packages in R, facilitated the prediction of disease prevalence, DALYs, and mortality through to the year 2035 (18). By incorporating age, period, and cohort effects, the BAPC model offers a nuanced framework for projecting future disease burdens (19).

### Statistics analysis

We expressed the incidence, prevalence, DALYs, and mortality rates as projections per 100,000 population, including their 95% uncertainty intervals (UI). Estimates of the EAPC included their 95% confidence intervals (CI). All analytical procedures and graphical representations were conducted using R Studio (Version 4.3.3 for Windows) and JD_GBDR (V2.37, Jingding Medical Technology Co., Ltd.). We considered *p*-values less than 0.05 as statistically significant.

## Results

### Global trends

**Figure 1:**
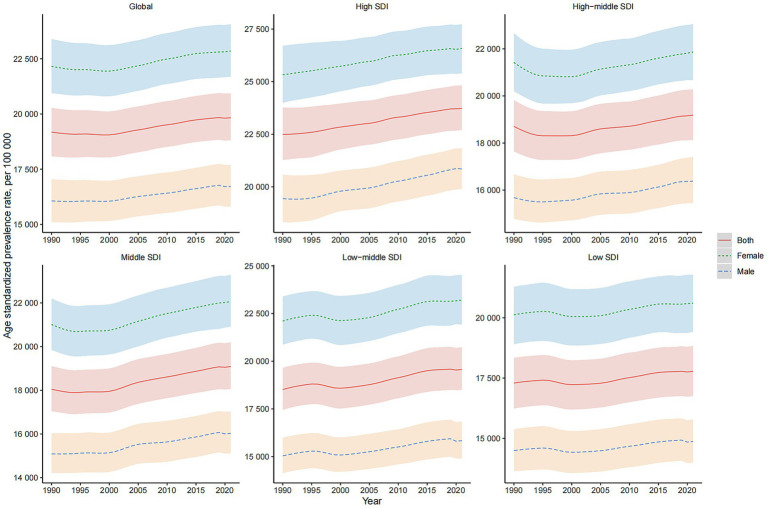
Global and SDI-stratified Prevalence Trends of MSDs from 1990 to 2021. This figure presents the prevalence trends of MSDs globally and across different levels of the SDI from 1990 to 2021. The trends are shown separately for both genders, highlighting the variations in MSD prevalence over time and among different SDI groups.

In 2021, the global prevalence of MSDs was estimated at 1,686.56 million cases (95% UI: 1,599.17 to 1,780.15 million), representing a 95% increase from 1990. The ASPR was 19.83 per 100,000 population, with an observed average annual increase of 0.150% since 1990 (Table 1; Figure 1). Regarding incidence, the number of new cases worldwide was 367.19 million (95% UI: 333.09 to 402.08 million) in 2021, marking a 71% increase since 1990. However, the ASIR was 4.53 per 100,000 population, showing an average annual decrease of 0.162% over the same period (Supplementary Table S1; Supplementary Figure S1). For DALYs attributed to MSDs, the global total reached 161.88 million (95% UI: 118.02 to 216.15 million) in 2021, reflecting an 88% increase from 1990. The ASDR was 1.91 per 100,000 population, with an average annual rise of 0.088% since 1990 (Supplementary Table S2; Supplementary Figure S2). Moreover, there were 118.50 million mortality globally attributable to MSDs in 2021 (95% UI: 103.13 to 128.55 million), a 103% increase since 1990. Despite this increase, the ASMR was 1.44 per 100,000 population, with an average annual decline of 0.265% during the same timeframe (Supplementary Table S3; Supplementary Figure S3).

**Table 1:** Age standardized prevalence rate (ASPR) of musculoskeletal disorders in 1990 and 2021, and estimated annual percentage change (EAPC) from 1990 to 2021 at the global and regional level.

Group	1990		2021		1990–2021	
	Prevalent cases ×1,000 (95% UI)	ASPRs per 100,000 (95% UI)	Prevalent cases ×1,000 (95% UI)	ASPRs per 100,000 (95% UI)	Total percent change (95% UI)	EAPC, % (95% CI)
Global	865073.99 (813102.88, 917749.06)	19.18 (18.08, 20.28)	1686561.52 (1599166.94, 1780146.35)	19.83 (18.81, 20.94)	0.95 (0.92, 0.98)	0.150 (0.130, 0.170)
SDI						
High	227753.51 (216117.18, 240227.72)	22.49 (21.28, 23.77)	363813.44 (348385.4, 379178.53)	23.72 (22.7, 24.82)	0.6 (0.57, 0.63)	0.191 (0.186, 0.196)
High-middle	196217.61 (184552.73, 208346.58)	18.71 (17.64, 19.82)	337089.86 (319246.11, 355491.8)	19.19 (18.13, 20.28)	0.72 (0.68, 0.75)	0.144 (0.114, 0.175)
Middle	238305.24 (223189.02, 253777.51)	18.05 (17.04, 19.11)	524541.98 (495535.98, 554819.46)	19.09 (18.06, 20.2)	1.2 (1.16, 1.25)	0.241 (0.217, 0.266)
Low-middle	148939.15 (139392.46, 159228.13)	18.52 (17.45, 19.66)	331961.62 (312545.82, 353057.76)	19.58 (18.5, 20.74)	1.23 (1.2, 1.26)	0.193 (0.162, 0.224)
Low	52981.48 (49315.63, 56630.19)	17.29 (16.24, 18.34)	127796.38 (119266.78, 136310.47)	17.78 (16.76, 18.83)	1.41 (1.39, 1.43)	0.100 (0.074, 0.125)
Regions						
Andean Latin America	4792.22 (4478.09, 5113.18)	18.08 (17.03, 19.17)	12380.81 (11706.83, 13136.09)	19.35 (18.34, 20.48)	1.58 (1.53, 1.63)	0.231 (0.219, 0.244)
Australasia	5229.06 (4951.18, 5536.86)	23.39 (22.12, 24.8)	9877.34 (9413.61, 10392.28)	24.01 (22.73, 25.44)	0.89 (0.84, 0.93)	0.109 (0.086, 0.132)
Caribbean	5035.8 (4727.92, 5330.86)	17.09 (16.12, 18.08)	9453.23 (8933.13, 9978.99)	18.12 (17.14, 19.14)	0.88 (0.84, 0.92)	0.195 (0.191, 0.199)
Central Asia	9327.68 (8749.64, 9971.2)	17.32 (16.24, 18.4)	16754.06 (15622.5, 17875.97)	18.34 (17.19, 19.49)	0.8 (0.77, 0.83)	0.205 (0.185, 0.225)
Central Europe	29046.73 (27168.77, 30992.03)	20.58 (19.2, 21.93)	35590.69 (33436.45, 37673.41)	21.23 (19.88, 22.59)	0.23 (0.2, 0.25)	0.106 (0.102, 0.109)
Central Latin America	23843.54 (22212.23, 25548.66)	20.76 (19.54, 22.04)	58122.39 (54835.2, 61545.23)	22.16 (20.93, 23.44)	1.44 (1.38, 1.49)	0.184 (0.166, 0.202)
Central Sub-Saharan Africa	5498.7 (5111.96, 5891.72)	17.22 (16.19, 18.32)	14456.58 (13400.88, 15487.8)	17.37 (16.33, 18.45)	1.63 (1.58, 1.68)	0.003(−0.017, 0.023)
East Asia	177345.86 (165865.17, 189261.22)	17 (15.99, 18.01)	355506.52 (335376.3, 374938.42)	17.48 (16.5, 18.49)	1 (0.94, 1.07)	0.214 (0.165, 0.265)
Eastern Europe	52691.21 (49377.86, 55972.58)	20 (18.7, 21.3)	59822.74 (56392.18, 63473.32)	20.33 (19.09, 21.58)	0.14 (0.12, 0.15)	0.126 (0.097, 0.155)
Eastern Sub-Saharan Africa	17270.44 (16043.32, 18514.99)	16.37 (15.36, 17.37)	43041.35 (40096.44, 46289.91)	16.96 (15.96, 17.97)	1.49 (1.47, 1.52)	0.121 (0.104, 0.138)
High-income Asia Pacific	47375.85 (44694.47, 50206.64)	23.84 (22.46, 25.27)	72214.68 (68709.1, 75657.7)	24.49 (23.16, 25.93)	0.52 (0.49, 0.56)	0.176 (0.129, 0.224)
High-income North America	79590.04 (75447.72, 83595.9)	25.14 (23.83, 26.43)	134363.48 (129922.32, 139001.9)	27.62 (26.64, 28.54)	0.69 (0.64, 0.74)	0.309 (0.293, 0.325)
North Africa and Middle East	43856.97 (40754.14, 47118.3)	18.88 (17.74, 20.05)	115845.07 (108384.35, 123549.39)	20.32 (19.15, 21.55)	1.64 (1.59, 1.69)	0.245 (0.235, 0.255)
Oceania	684.25 (639.08, 733.14)	16.4 (15.46, 17.39)	1729.82 (1615.43, 1849.54)	16.94 (15.96, 17.99)	1.53 (1.49, 1.57)	0.099 (0.091, 0.108)
South Asia	146674.31 (137572.79, 156930.5)	19 (17.89, 20.19)	345207.87 (324372.75, 368677.02)	20.14 (19, 21.41)	1.35 (1.32, 1.39)	0.218 (0.161, 0.276)
Southeast Asia	53645.73 (50112.73, 57326.36)	16.03 (15.12, 16.98)	124699.57 (117426.31, 132553.29)	17.22 (16.29, 18.26)	1.32 (1.28, 1.37)	0.239 (0.236, 0.243)
Southern Latin America	11531.57 (10887.1, 12244.98)	24.26 (22.94, 25.73)	19787.95 (18726.76, 20862.53)	25.15 (23.75, 26.58)	0.72 (0.68, 0.75)	0.129 (0.096,0.163)
Southern Sub-Saharan Africa	6001.56 (5615.76, 6402.45)	17.41 (16.42, 18.38)	12544.41 (11776.54, 13303.92)	18.16 (17.16, 19.15)	1.09 (1.06, 1.12)	0.159 (0.152,0.167)
Tropical Latin America	26795.13 (24982.91, 28681.84)	22.34 (21.05, 23.67)	57930.35 (54578.45, 61323.85)	22.62 (21.33, 23.97)	1.16 (1.11, 1.21)	−0.046 (−0.097, 0.005)
Western Europe	98977.99 (93669.17, 104566.14)	20.52 (19.38, 21.76)	135587.64 (128545.46, 142549.96)	21 (19.86, 22.25)	0.37 (0.35, 0.39)	0.083 (0.073, 0.093)
Western Sub-Saharan Africa	19859.35 (18548.45, 21183.77)	16.96 (15.96, 17.93)	51644.98 (48152.19, 55185.68)	17.65 (16.61, 18.63)	1.6 (1.58, 1.62)	0.132 (0.124,0.139)

ASPR = age standardized prevalence rate; EAPC = estimated annual percentage change; SDI = socio-demographic index; 95% UI = 95% uncertainty interval; 95% CI = 95% confidence interval.

Across all metrics, women exhibited higher age-standardized rates and total case numbers of MSDs compared to men. Furthermore, the trends in ASPR, ASIR, ASDR, and ASMR over the past three decades varied significantly across different regions characterized by varying levels of the SDI and between genders (Figure 1; Supplementary Figures S1–S3).

### Regional trends

In 2021, an analysis of the 21 GBD regions revealed distinct patterns in the prevalence, incidence, and impact of MSDs. The regions of Australasia, the Caribbean, and High-income Asia Pacific reported the highest prevalence of MSDs cases, as detailed in Table 1. Conversely, the highest incidence rates were observed in East Asia, South Asia, and Central Asia (Supplementary Table S1). Additionally, the same top three regions—Australasia, the Caribbean, and High-income Asia Pacific—recorded the greatest number of DALYs attributed to MSDs (Supplementary Table S2). The highest mortality rates were noted in High-income North America, Andean Latin America, and High-income Asia Pacific (Supplementary Table S3).

Among these regions in 2021, High-income North America (27.62), Central Southern Latin America (25.15), and Australasia (24.01) exhibited the highest ASPR for MSDs per 100,000 population. In stark contrast, the lowest ASPRs were reported in Oceania (16.94), Eastern Sub-Saharan Africa (16.96), and Southeast Asia (17.22) (Table 1). Similarly, the highest ASIR for MSDs per 100,000 population were recorded in Central Europe (6.32), Eastern Europe (5.9), and Australasia (5.82), while the lowest ASIRs were observed in Southeast Asia (3.60), East Asia (3.65), and Andean Latin America (3.71) (Supplementary Table S1). Regarding the ASDR for MSDs in 2021, High-income North America (2.76), Southern Latin America (2.56), and Australasia (2.42) reported the highest figures. The regions with the lowest ASDR were Oceania (1.54), East Asia (1.59), and Eastern Sub-Saharan Africa (1.61) (Supplementary Table S2). Furthermore, the highest ASMR for MSDs were seen in South Asia (2.52), Central Latin America (2.47), and Southern Sub-Saharan Africa (2.09), while the lowest rates were recorded in Oceania (0.35), Eastern Sub-Saharan Africa (0.55), and Central Asia (0.62) (Supplementary Table S3). Supplementary Figures S4-S7 delineate the distribution of ASPR, ASIR, ASDR, and ASMR for MSDs by sex across all GBD regions in 2021.

From 1990 to 2021, the EAPC of ASPR exhibited the most significant increases in Central Asia (6.138%), Eastern Europe (1.271%), and Western Sub-Saharan Africa (0.704%). In contrast, substantial decreases were noted in Central Europe (−2.015%), High-income Asia Pacific (−1.590%), and High-income North America (−1.142%) (Table 1). A similar pattern of changes was observed for ASIR, ASDR, and ASMR during the same period, as detailed in Tables S1-S3. The EAPC of ASPR, ASIR, ASDR, and ASMR for MSDs by sex across all regions is depicted in Supplementary Figures S8–S11.

### National trends

**Figure 2:**
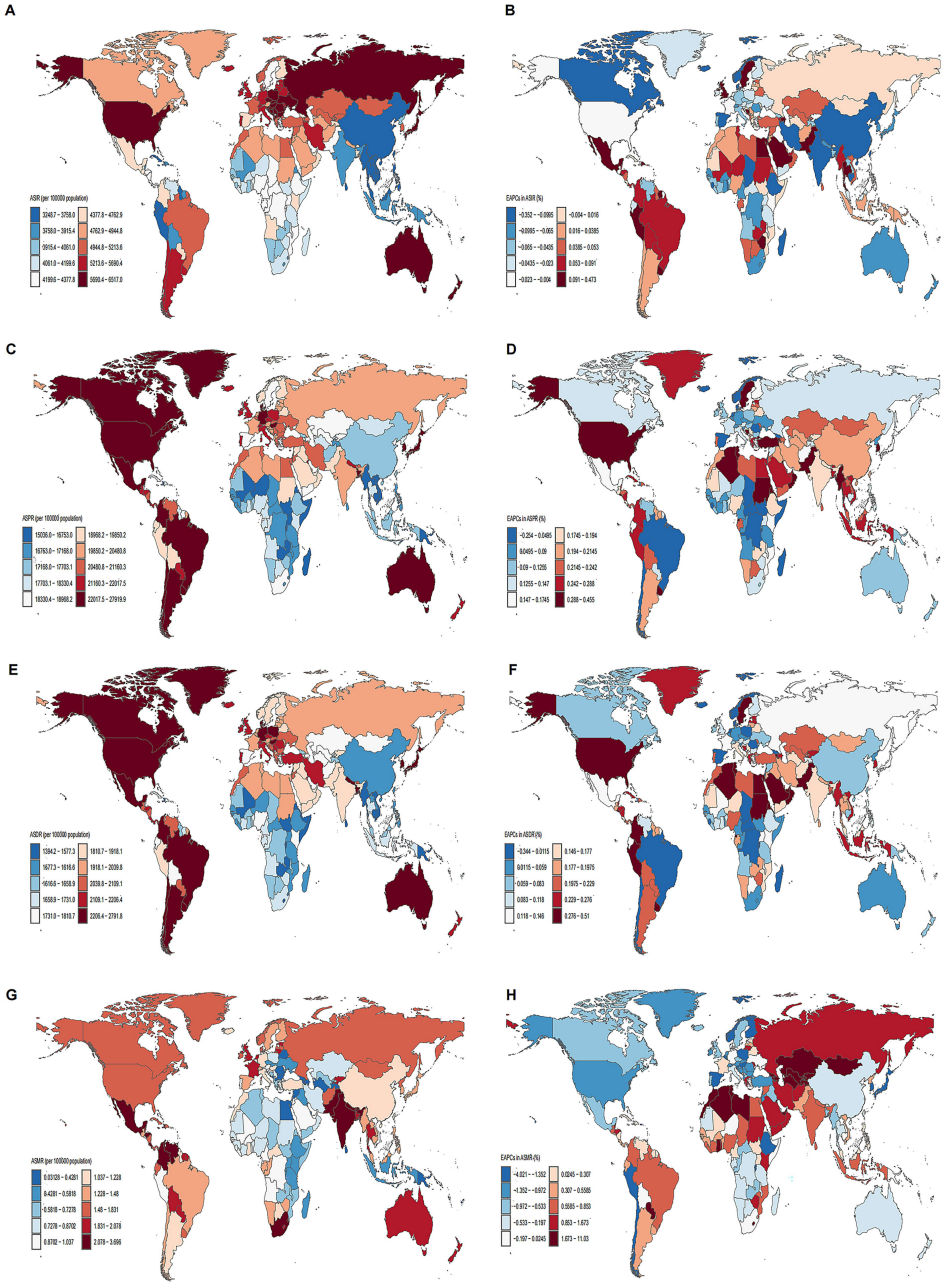
Maps of ASRs of MSDs in 2021 and EAPC from 1990 to 2021 in 204 countries and territories. **(A)** ASIR. **(B)** EAPC in ASIR. **(C)** ASPR. **(D)** EAPC in ASPR. **(E)** ASDR. **(F)** EAPC in ASDR. **(G)** ASMR. **(H)** EAPC in ASMR. Illustrating the global geographical distribution of ASPR, ASIR, and ASMR of MSDs in 2021. The maps are color-coded based on different rate ranges, providing a visual representation of the regional disparities in MSDs burden.

In 2021, the ASPR of MSDs varied significantly, ranging from 15,036.01 to 27,919.91 per 100,000 individuals. The United States reported the highest rate at 27,919.91, followed closely by Chile (25,281.83) and Argentina (25,109.85). In contrast, the lowest rates were observed in Eritrea at 15,036.01, Burundi at 15,279.79, and Timor-Leste at 15,706.01 (Figure 2C; Supplementary Table S4). The ASIR spanned from 3,248.67 to 6,517.02 per 100,000 population, with Hungary (6,517.02), Poland (6,515.74), and Czechia (6,341.32) recording the highest rates, whereas Myanmar (3,248.67), the Maldives (3,261.54), and Timor-Leste (3,325.12) exhibited the lowest (Figure 2A; Supplementary Table S4). The ASDR was highest in the United States (2,791.85), Japan (2,575.22), and Chile (2,572.90), with the lowest values found in Eritrea (1,394.16), Burundi (1,449.73), and Timor-Leste (1,461.54) (Figure 2E; Supplementary Table S4). The ASMR varied from a minimal 0.01 to a maximum of 3.71 per 100,000 population, with the highest rates in Saint Kitts and Nevis (3.71), the Bahamas (3.70), and Barbados (3.55), and the lowest in the Cook Islands (0.01), Azerbaijan (0.03), and Belarus (0.06) (Figure 2G, Supplementary Table S4).

From 1990 to 2021, the ASPR exhibited the largest increases in Pakistan (0.46%), Taiwan (0.43%), and Oman (0.42%), with the most significant decreases in Burundi (−0.25%), Denmark (−0.17%), and Chile (−0.10%) (Figure 2D; Supplementary Table S4). The ASIR increased most notably in Sweden (0.47%), Pakistan (0.35%), and Taiwan (0.32%), and decreased most prominently in Denmark (−0.35%), India (−0.31%), and China (−0.18%) (Figure 2B; Supplementary Table S4). For ASDR, the largest increases were seen in Taiwan (0.57%), Pakistan (0.51%), and Oman (0.43%), while the most substantial declines occurred in Burundi (−0.34%), Denmark (−0.27%), and Spain (−0.25%) (Figure 2F; Supplementary Table S4). The ASMR rose sharply in Georgia (9.97%), Armenia (9.95%), and Kazakhstan (9.79%), but significantly fell in Spain (−4.02%), Singapore (−3.23%), and Poland (−3.20%) (Figure 2H; Supplementary Table S4). Supplementary Table S4 further summarizes the overall case percentages and the EAPC for ASPR, ASIR, ASDR, and ASMR across 204 countries and regions.

### Age and sex patterns

**Figure 3:**
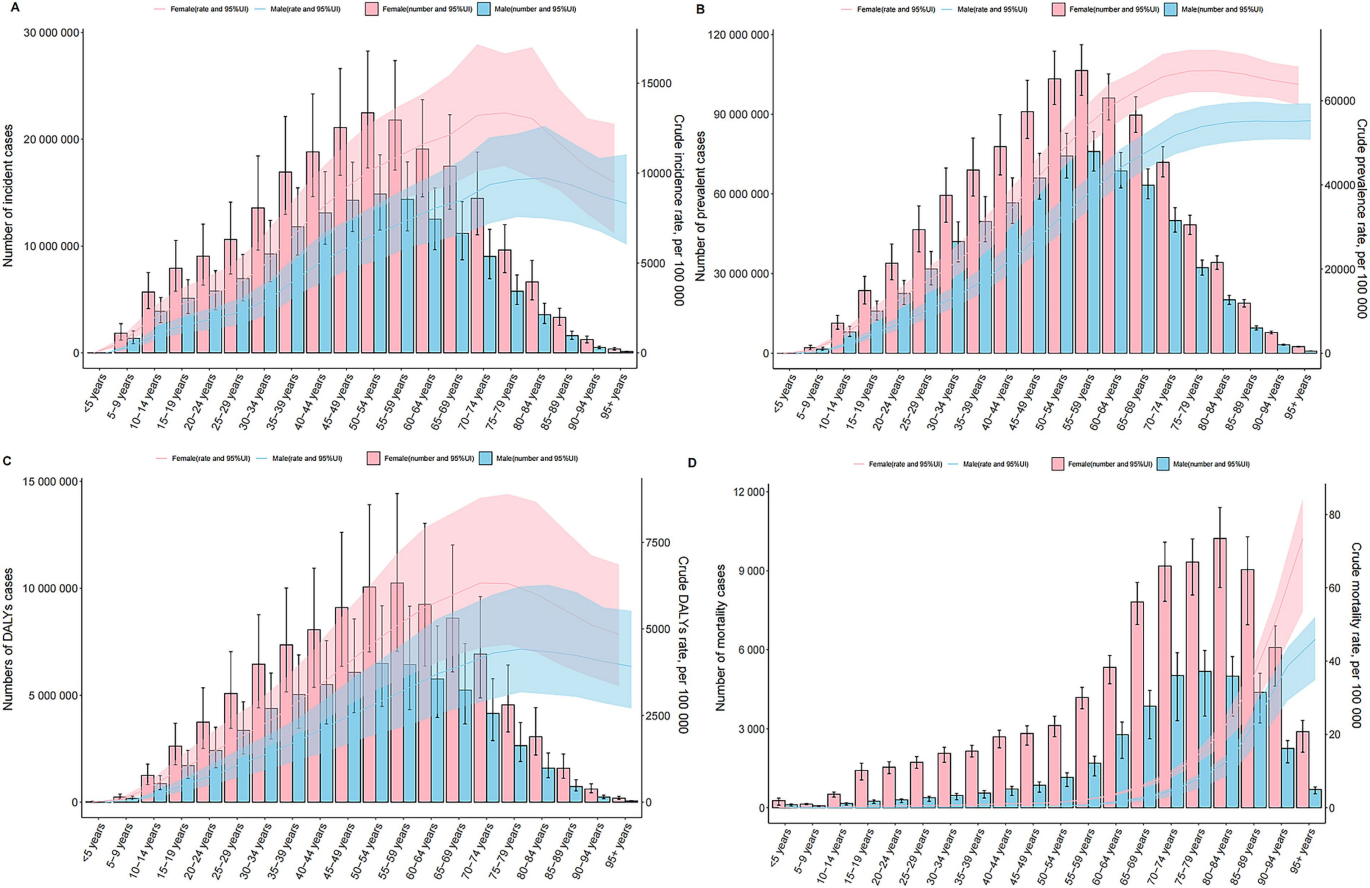
Age and Sex Patterns of MSDs at the Global Level. Depicting the incidence, prevalence, DALYs, and mortality related to MSDs by age and sex. **(A)** Incidence. **(B)** Prevalence. **(C)** DALYs. **(D)** Mortality. Both the rates and numbers are presented, showing the differences in MSDs occurrence and impact between males and females across different age cohorts globally.

In 2021, the global prevalence of MSDs began increasing in individuals aged 5–9 years, peaked in those aged 85–89 years, and then demonstrated a slight decline. The age group with the highest number of prevalent cases was 55–59 years. Across all age groups, both the prevalence and the total number of MSDs cases were consistently higher in females than in males (Figure 3B). The highest global incidence of MSDs was observed in the 75–79 years age group. Among females, the peak incidence occurred within the same age group, whereas among males, it peaked slightly later, in the 80–84 years age group. Females exhibited a higher incidence rate across all age groups (Figure 3A). The global burden of DALYs due to MSDs was also highest in the 75–79 years age group, with a subsequent decline noted in older age groups. Females reported higher DALY rates than males in every age category (Figure 3C). Moreover, global mortality rates due to MSDs increased with age, reaching a peak in the 80–84 years age group (Figure 3D).

### Association with the SDI

**Figure 4:**
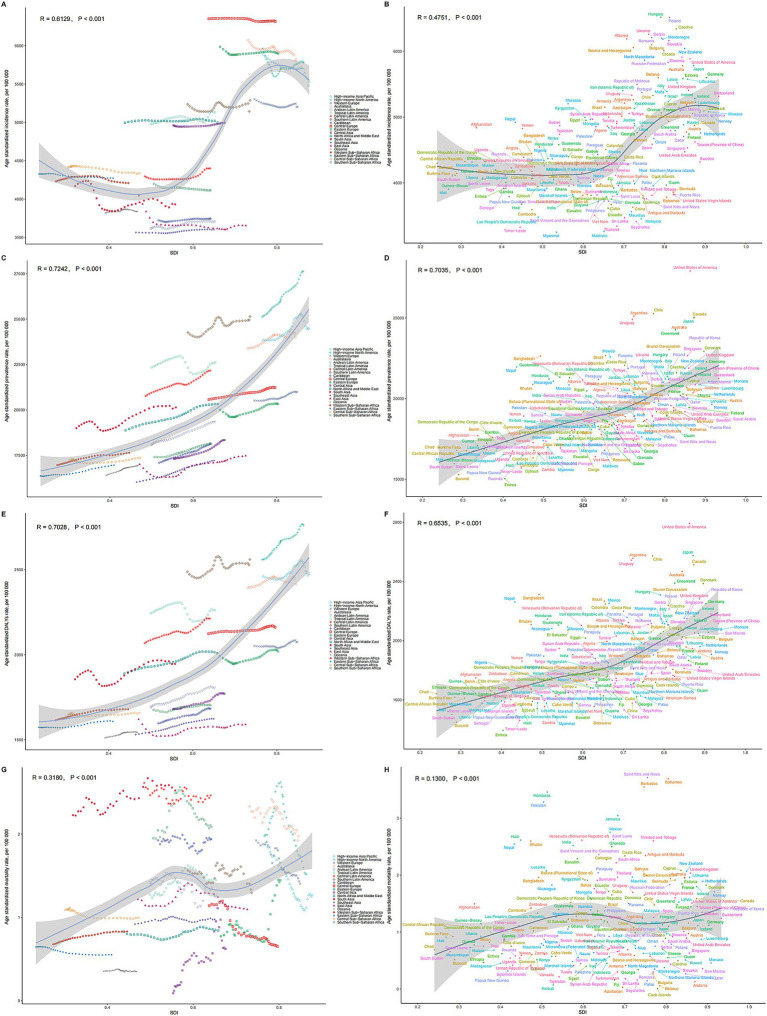
Relationships between SDI and ASRs of MSDs. **(A)** SDI and ASIR among different regions. **(B)** SDI and ASIR among different countries. **(C)** SDI and ASPR among different regions. **(D)** SDI and ASIR among different countries. **(E)** SDI and ASDR among different regions. **(F)** SDI and ASDR among different countries. **(G)** SDI and ASMR among different regions. **(H)** SDI and ASMR among different countries. The scatter plots show the trends and correlations, with regression lines and corresponding R and *p* values, indicating the strength and significance of the relationships among different regions or nations. Relationship was observed between the SDI and the ASPR of MSDs.

At the regional level, a U-shaped relationship was discerned between the SDI and the ASPR of MSDs from 1990 to 2021. Initially, an increase in the SDI corresponded with a decline in the ASPR, which reached its nadir at an SDI of approximately 0.5, before ascending again. Notably, Southern Latin America exhibited a significantly higher ASPR than anticipated based on their SDI, whereas East Asia consistently demonstrated much lower than expected ASPR (Figure 4C). Comparable patterns were observed for the ASIR, ASDR, and ASMR in relation to SDI (Figures 4A,E,G).

At the national level in 2021, the ASPR of MSDs generally rose with an increase in the SDI. The United States exhibited a much higher than expected burden of MSDs, whereas Eritrea showed a considerably lower than anticipated burden (Figure 4D). Similar national-level patterns were observed for ASIR, ASDR, and ASMR in relation to SDI (Figures 4B,F,H).

### Predictive analysis

**Figure 5:**
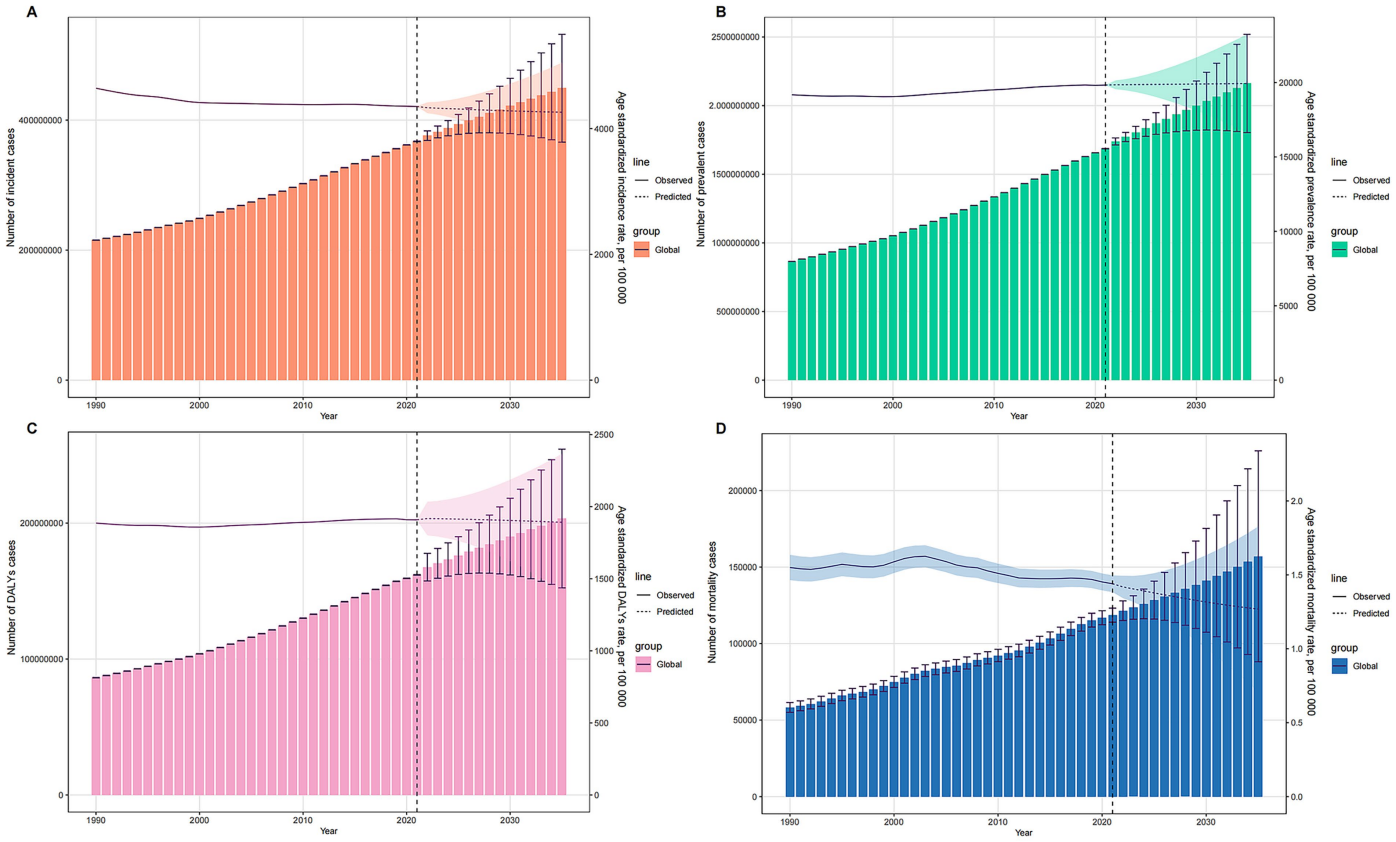
Projections of MSDs Burden from 1990 to 2035 Using BAPC Model. **(A)** Incidence cases and ASIR. **(B)** Prevalence cases and ASPR. **(C)** DALYs cases and ASDR. **(D)** Mortality cases and ASMR. Presenting the projected trends of the number of incident cases, prevalent cases, DALYs cases, and mortality cases of musculoskeletal disorders from 1990 to 2035. The observed and predicted values of ASRs (ASIR, ASPR, ASDR, and ASMR) are also shown, demonstrating the expected changes in MSD burden over time.

The predictions for the number of cases and ASRs of prevalence, incidence, DALYs, and mortality associated with MSDs through 2035 are detailed in Figure 5. It is anticipated that the global totals for incidence, prevalence, DALYs, and mortality will incrementally rise, reaching estimates of 449 million, 2,161 million, 203 million, and 157 million, respectively, by 2035. In contrast, the ASRs for these indicators are forecasted to experience an annual decline through the same period (Figures 5A–D). By 2035, the ASIR, ASPR, ASDR, and ASMR are projected to decrease to 4,259.84; 19,936.66; 1,891.66; and 1.27 per 100,000 population, respectively.

### Frontier analysis

**Figure 6:**
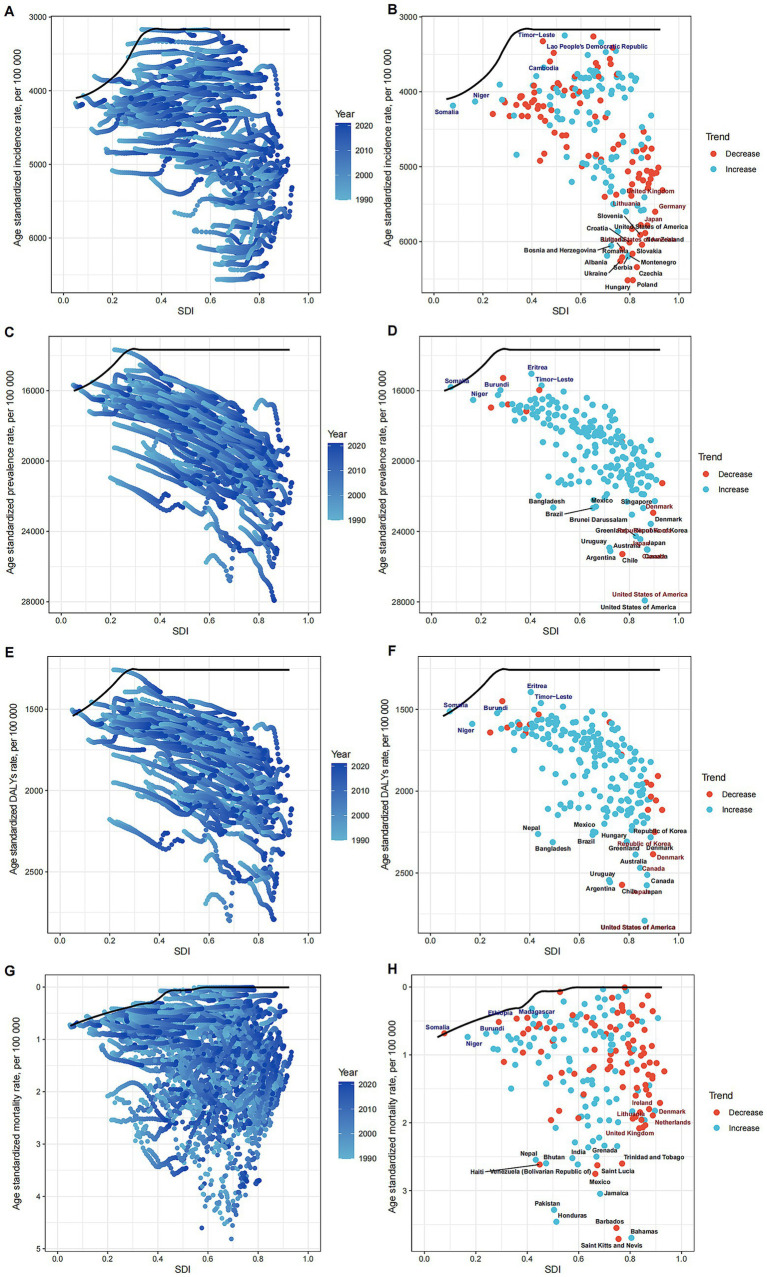
Frontier Analysis of MSDs Burden in Relation to SDI. Illustrating the frontier analysis results of musculoskeletal disorders burden in relation to the SDI. **(A)** SDI and ASIR among different regions. **(B)** SDI and ASIR among different countries. **(C)** SDI and ASPR among different regions. **(D)** SDI and ASIR among different countries. **(E)** SDI and ASDR among different regions. **(F)** SDI and ASDR among different countries. **(G)** SDI and ASMR among different regions. **(H)** SDI and ASMR among different countries. The figures show the differences between the observed and achievable minimum ASRs (incidence, prevalence, DALYs, and mortality rates) across various countries and regions, highlighting the potential for improvement in MSD burden management.

Utilizing data spanning from 1990 to 2021, we conducted a frontier analysis using ASIR, ASPR, ASDR, and ASMR in relation to the SDI to assess potential improvements in managing the burden of MSDs while considering varying levels of national and regional development (Figure 6). The frontier curve illustrates the lowest (optimal) ASR observed for each specific SDI level, and the deviation from this curve, termed the effective difference, measures the discrepancy between a country or region’s observed ASR and the achievable ASR under ideal conditions. For 2021, the effective difference for ASPR was calculated for each country and region based on their respective SDI levels (Figure 6D). The results showed that the effective difference generally decreased as the SDI increased, with the variance appearing to stabilize at higher SDI levels. Among the 15 countries and regions identified with the largest scope for improvement, the effective difference ranged from 117.40 to 9,887.10. These included the Republic of Korea, Brunei Darussalam, Denmark, Singapore, Brazil, Bangladesh, Mexico, Hungary, Germany, Colombia, Nepal, the United Kingdom, Panama, New Zealand, and Poland. Notably, countries and regions with lower SDIs such as Somalia, Niger, Nepal, Solomon Islands, and The Gambia were highlighted as performing relatively well despite their limited resources. Moreover, countries like Niger and Burundi, despite their lower SDIs, have demonstrated effective management of disease burden. The frontier analyses for ASIR (Figures 6A,B), ASDR (Figures 6E,F), and ASMR (Figures 6G,H) further corroborated the relationship between SDI and the potential for reducing the MSDs burden.

## Discussion

This study conducted a comprehensive analysis of the epidemiological characteristics and trends of MSDs at global, regional, and national levels from 1990 to 2021. The findings indicate that over the past three decades, the prevalence, incidence, DALYs, and mortality attributable to MSDs have all increased worldwide. However, the ASRs have generally declined during this period. Regionally, Australasia, the Caribbean, and the High-income Asia Pacific regions experienced the greatest MSDs burden. Nationally, the United States, Chile, and Argentina reported the highest prevalence and disability burdens associated with MSDs, whereas Eritrea, Burundi, and Timor-Leste had the lowest. The data also showed that women experienced higher prevalence, incidence, DALY rates, and mortality rates of MSDs compared to men. Furthermore, the study identified a U-shaped relationship between the MSDs burden and national development levels, with the lowest burden occurring at medium development levels. Predictive analysis suggests that while the absolute number of MSDs cases is expected to continue rising through 2035, the standardized rates are projected to decline. Additionally, frontier analysis highlighted variations in the MSDs burden across different countries.

This study utilized the updated GBD 2021 data to conduct an exhaustive and systematic analysis of the epidemiological characteristics of MSDs across global, regional, and national contexts. The findings largely align with those reported in the GBD 2017 (20) and GBD 2019 (21) studies, confirming that MSDs continues to be a primary cause of disability worldwide, disproportionately affecting women more than men. The global trend analysis revealed that the number of MSDs cases worldwide escalated to 1.686 billion in 2021, marking a 95% increase since 1990. This substantial rise likely reflects the compounded impact of an aging global population and advancements in medical diagnostics. The analysis of regional disparities indicated that the age-standardized prevalence of MSDs was highest in affluent, developed regions such as North America and Australasia, whereas it was lowest in Southeast Asia and sub-Saharan Africa. These regional variances can be attributed to several factors: (1) disparities in economic development and medical resources, which influence the levels of disease diagnosis and treatment (6); (2) the heterogeneous geographic distribution of population age structures (5); and (3) differences in lifestyle and occupational characteristics across regions (22). Further analysis of country-specific MSDs burdens in 2021 demonstrated significant variability in age-standardized prevalence rates. The United States, Chile, and Argentina exhibited the highest rates, whereas Eritrea, Burundi, and Timor-Leste reported the lowest. Generally, age-standardized metrics of MSDs, such as prevalence, incidence, and DALY rates, tended to increase with a country’s SDI (23). Nonetheless, notable deviations were observed at the individual country level; for instance, the MSDs burden in the United States significantly exceeded expectations for its SDI level, while Eritrea’s burden was considerably below what was anticipated. From 1990 to 2021, the trends in MSDs indicators exhibited notable changes in certain countries. Pakistan, Taiwan (Province of China), and Oman experienced the most pronounced increases in prevalence rates, whereas Burundi, Denmark, and Chile saw the most significant reductions. These findings suggest that, even among countries with similar developmental statuses, there exist variations in the approaches to MSDs prevention and management.

Women exhibit higher rates of MSDs compared to men across various indicators including prevalence, incidence, DALYs, and mortality. This disparity may be attributable to several factors specific to women such as physiological conditions including osteoporosis and joint degeneration, psychological factors like anxiety and depression, and a greater burden of physical labor both in domestic and professional contexts (24, 25). Consequently, gender differences play a crucial role in the epidemiology of MSDs. Implementing targeted interventions that address these factors could help reduce the MSDs disparity between genders. The age distribution of MSDs burden shows an initial increase followed by a decrease, peaking in the 75–79 age group. This pattern underscores the link between MSDs burden and population aging, suggesting that preventive and interventional strategies should primarily target the middle-aged and older adult populations (26).

An analysis of the relationship between MSDs and the SDI reveals a U-shaped correlation with standardized incidence rates, mortality rates, and DALYs. Rates are lowest at an intermediate level of economic development. This observation indicates that economic status significantly influences MSDs outcomes. Regions with low economic levels suffer from inadequate health resources and poor prevention and treatment capabilities, while highly developed areas may face elevated risks associated with industrialization and an aging population. The pronounced MSDs burden in affluent regions may stem from high levels of industrialization and shifts in physical labor and lifestyle patterns (27). It is imperative that regional strategies for MSDs prevention and management be tailored to reflect local conditions.

Projections for MSDs burden up to 2035 indicate a declining trend in ASIR, prevalence, DALY rates, and mortality rates, suggesting that current preventive measures are effective. However, there is a need for further enhancement to address the growing total burden of the disease. Despite the downward trends in ASRs, the overall burden of MSDs continues to rise. This increase may be driven by factors such as global population aging, the accumulation of disease risk associated with extended life expectancy, and enhanced medical diagnostic capabilities in developing countries, which allow for more comprehensive case detection and reporting. ^4^ Additionally, lifestyle changes such as decreased physical activity and increasing obesity rates are also significant contributors to the rising MSDs burden (28, 29). As industrialization accelerates, particularly in developing countries, these risk factors are becoming more prevalent and warrant significant attention (28).

The analysis of frontiers demonstrated significant disparities in the burden of MSDs across different countries and regions, particularly in those with a higher SDI, where the control of MSDs burden appeared more effective. Our study, which spanned from 1990 to 2021, revealed that with increasing SDI, the gap between the observed ASIR and the optimal ASIR narrowed. This finding underscores the strong link between economic development and health outcomes and offers valuable insights for countries addressing MSDs. In the 2021 data, the gap in the 15 countries and regions with the greatest potential for improvement varied widely, ranging from 117.40 to 9,887.10, highlighting significant opportunities for enhancing MSDs management. Notably, countries such as South Korea, Singapore, and Germany, despite their relatively high SDI, still exhibited considerable gaps, suggesting further room for improvement in their health policies and allocation of medical resources. Conversely, countries with lower SDI, like Niger and Burundi, demonstrated commendable control over their disease burden despite limited resources. This indicates that the effectiveness of health interventions is not solely reliant on economic prosperity; well-crafted public health policies and community interventions also play a critical role in environments with scarce resources. Further analysis of frontier data, including ASIR, ASDR, and ASMR, consistently correlated with SDI, reinforcing the role of economic development in reducing MSDs burden potential. These findings emphasize the necessity for health policy makers at both global and national levels to consider the interplay between economic status and disease burden when creating sustainable health improvement strategies. Looking ahead, it is crucial to continue monitoring countries that have demonstrated exemplary control of MSDs burden despite low SDI, and to distill lessons from their success to inform strategies in other nations.

Current research often focuses on common socio-economic factors. Future studies should broaden their scope to investigate the impact of lifestyle, psychological, and genetic factors on MSDs incidence to gain a more comprehensive understanding of its etiology. Given the diversity in pathogenesis, population characteristics, and outcomes among MSDs subtypes, such as osteoarthritis, low back pain, and periarthritis, it is essential to delve deeper into the epidemiological traits of these subtypes to craft targeted interventions. Additionally, the prevalent use of cross-sectional study designs has limitations in capturing the dynamic evolution of MSDs prevalence. Longitudinal studies, such as cohort studies, should be employed to track the progression and determinants of MSDs in specific populations or regions over time (30).

This study employed a basic linear extrapolation method for forecasting purposes. Future research should explore more sophisticated predictive methodologies, such as time series analysis and system dynamics models, to enhance the accuracy of predictions regarding the future prevalence trends of MSDs. Broadly speaking, subsequent epidemiological investigations into MSDs should adopt a multidimensional approach. These studies should not only focus on understanding the pathogenesis and risk factors but also emphasize the importance of monitoring dynamic trends and improving the precision of predictions. This comprehensive approach is essential for developing more effective prevention and intervention strategies.

The current study also encountered several limitations. First, despite the extensive coverage of the GBD data, gaps and instances of poor data quality were present in some regions and countries, potentially compromising the accuracy and representativeness of the findings. Second, the analysis was restricted to the overall burden of MSDs, with inadequate attention given to the specific characteristics and determinants of different MSDs subtypes. Future studies should place greater emphasis on exploring the variations among these subtypes. Lastly, the predictive analysis relied on a simplistic linear extrapolation method, which may not adequately reflect the complexities of the MSDs prevalence trends. Future research should consider employing more complex predictive models to better capture these nuances.

## Conclusion

The global burden of MSDs has escalated significantly over the past three decades, reaching approximately 1.69 billion prevalent cases in 2021—an increase of 95% since 1990. Concurrently, there has been a substantial rise in the total number of incident cases, DALYs, and mortality. Despite this increasing overall burden, ASRs for incidence, prevalence, DALYs, and mortality have generally exhibited decreasing trends worldwide. Notable regional and national disparities were observed; high-income regions such as Australasia reported the highest burdens, whereas low-income regions like Oceania reported the lowest. Additionally, women consistently displayed higher ASRs compared to men. Frontier analysis underscored a U-shaped relationship between the SDI and age-standardized MSDs rates, pinpointing opportunities for enhancements, particularly in high-SDI countries. Projections suggest that while the total number of MSDs cases, DALYs, and mortality is anticipated to continue to rise by 2035, ASRs are expected to decline. This dynamic underscores the imperative for targeted strategies to manage the growing absolute burden effectively. In conclusion, the global burden of MSDs remains substantial and is projected to increase further, highlighting the critical need for tailored interventions to effectively address this significant public health challenge.

## Data Availability

The original contributions presented in the study are included in the article/Supplementary material, further inquiries can be directed to the corresponding author/s.
