# Supplementary material for: Global, regional, and national burden of musculoskeletal disorders, 1990–2021: an analysis of the global burden of disease study 2021 and forecast to 2035
Source: Front Public Health. 2025 Aug 1;13:1562701. doi: 10.3389/fpubh.2025.1562701 (PMC12354483; doi:10.3389/fpubh.2025.1562701)
Supplement: Supplementary file 1 [file Table_1.doc]

**Table S1** Age standardized incidence rate (ASIR) of musculoskeletal disorders in 1990 and 2021, and estimated annual percentage change (EAPC) from 1990 to 2021 at the global and regional level.

| Group | **1990** | | **2021** | | **1990-2021** | |
| --- | --- | --- | --- | --- | --- | --- |
|  | **Incident cases,**  **×1000 (95% UI)** | **ASIRs**  **per 100 000**  **(95% UI)** | **Incident cases,**  **×1000 (95% UI)** | **ASIRs**  **per 100 000**  **(95% UI)** | **Total percent change**  **(95% UI)** | **EAPC, %,**  **(95% CI)** |
| Global | 215339.15(194696.72,237685.15) | 4.64(4.2,5.1) | 367193.43(333085.12,402083.08) | 4.35(3.96,4.76) | 0.71(0.67, 0.74) | -0.162(-0.196,-0.129) |
| SDI |  |  |  |  |  |  |
| High | 55643.09(50462.75,61172.59) | 5.61(5.08,6.16) | 78388.14(71420.03,85006.51) | 5.39(4.94,5.88) | 0.41(0.38, 0.44) | -0.085(-0.111,-0.059) |
| High-middle | 51737.42(46635.28,57150.45) | 4.89(4.41,5.38) | 75796.64(68172.79,82973.85) | 4.49(4.08,4.91) | 0.47(0.43, 0.5) | -0.211(-0.253,-0.170) |
| Middle | 57503.06(51859.85,63640.71) | 4.15(3.75,4.56) | 108378.08(98402.4,118969.67) | 3.98(3.63,4.36) | 0.88(0.83, 0.94) | -0.057(-0.100,-0.014) |
| Low-middle | 36099.46(32508.3,40160.49) | 4.25(3.84,4.69) | 72038.5(64937.4,79438.75) | 4.15(3.75,4.57) | 1(0.96, 1.03) | -0.088(-0.140,-0.036) |
| Low | 14115.54(12717.87,15673.43) | 4.29(3.87,4.73) | 32261.29(29086.64,35791.17) | 4.17(3.77,4.58) | 1.29(1.27, 1.3) | -0.112(-0.137,-0.086) |
| Regions |  |  |  |  |  |  |
| Andean Latin America | 1039.32(943.91,1143.4) | 3.61(3.28,3.96) | 2404.87(2187.25,2630.33) | 3.71(3.38,4.05) | 1.31(1.25, 1.38) | 0.099(0.082,0.116) |
| Australasia | 1340.48(1217.23,1487.54) | 6.07(5.53,6.75) | 2257.4(2032.66,2479.7) | 5.82(5.27,6.39) | 0.68(0.63, 0.73) | -0.079(-0.099,-0.058) |
| Caribbean | 1146.19(1039.1,1260.04) | 3.72(3.37,4.08) | 1923.46(1748.9,2098.39) | 3.75(3.41,4.09) | 0.68(0.64, 0.72) | 0.039(0.031,0.046) |
| Central Asia | 2780.09(2507.47,3080.19) | 4.96(4.5,5.48) | 4655.36(4195.9,5175.28) | 5(4.52,5.53) | 0.67(0.64, 0.71) | 0.029(0.021,0.037) |
| Central Europe | 8841.16(7944.82,9787.67) | 6.35(5.72,7.03) | 9935.48(8880.2,10892.03) | 6.32(5.69,6.99) | 0.12(0.1, 0.16) | -0.026(-0.032,-0.021) |
| Central Latin America | 5391.59(4882.41,5963.82) | 4.3(3.89,4.74) | 11576.04(10486.21,12751.18) | 4.4(3.98,4.84) | 1.15(1.08, 1.22) | 0.079(0.048,0.110) |
| Central Sub-Saharan Africa | 1535.15(1381.37,1703.22) | 4.42(3.97,4.87) | 3958.03(3584.89,4406.09) | 4.34(3.93,4.77) | 1.58(1.53, 1.63) | -0.077(-0.092,-0.062) |
| East Asia | 43610.54(39288.2,48232.52) | 4.05(3.66,4.44) | 71531.14(64649.25,78022.78) | 3.65(3.33,3.98) | 0.64(0.57, 0.71) | -0.172(-0.248,-0.096) |
| Eastern Europe | 15430.64(13823.98,17029.15) | 5.98(5.39,6.6) | 16442.97(14665.02,18034.73) | 5.9(5.31,6.49) | 0.07(0.04, 0.09) | 0.002(-0.013,0.017) |
| Eastern Sub-Saharan Africa | 4984(4499.27,5540.04) | 4.33(3.91,4.77) | 11861.46(10704.32,13165) | 4.27(3.86,4.71) | 1.38(1.36, 1.4) | -0.051(-0.057,-0.045) |
| High-income Asia Pacific | 11548.91(10456.93,12723.04) | 5.89(5.31,6.5) | 14811.05(13368.28,16155.71) | 5.53(5.01,6.09) | 0.28(0.24, 0.33) | -0.138(-0.166,-0.110) |
| High-income North America | 18561.65(16810.71,20480.8) | 5.96(5.43,6.55) | 27161.68(25059.25,29222.67) | 5.78(5.37,6.24) | 0.46(0.41, 0.52) | -0.014(-0.072,0.043) |
| North Africa and Middle East | 12586.64(11386.03,13868.07) | 5(4.51,5.48) | 29272.46(26542.14,32401.04) | 5(4.5,5.48) | 1.33(1.27, 1.38) | 0.009(0.000,0.017) |
| Oceania | 171.75(154.2,190.23) | 3.82(3.46,4.2) | 415.7(374.3,460.32) | 3.84(3.48,4.22) | 1.42(1.37, 1.47) | 0.023(0.010,0.036) |
| South Asia | 33602.15(30187.53,37310.93) | 4.17(3.76,4.61) | 68149.24(61465.05,75400.45) | 3.93(3.55,4.32) | 1.03(0.99, 1.07) | -0.212(-0.317,-0.106) |
| Southeast Asia | 12495.76(11266.36,13795.02) | 3.56(3.23,3.91) | 26123.65(23665.49,28853.07) | 3.6(3.27,3.96) | 1.09(1.03, 1.15) | 0.052(0.043,0.061) |
| Southern Latin America | 2508.07(2266.04,2771.01) | 5.24(4.74,5.79) | 4079.11(3695.89,4493.82) | 5.29(4.78,5.83) | 0.63(0.59, 0.67) | 0.038(0.009,0.067) |
| Southern Sub-Saharan Africa | 1542.26(1399.41,1694.57) | 4.2(3.8,4.58) | 2940.93(2664.91,3244.32) | 4.11(3.72,4.5) | 0.91(0.87, 0.94) | -0.046(-0.054,-0.039) |
| Tropical Latin America | 6329.55(5690.18,7028.66) | 4.98(4.49,5.52) | 12925.79(11648.72,14286.35) | 5.13(4.63,5.66) | 1.04(0.97, 1.11) | 0.061(0.035,0.086) |
| Western Europe | 24550.24(22223.4,26888.97) | 5.3(4.82,5.82) | 31284.58(28166.12,34224.79) | 5.2(4.7,5.73) | 0.27(0.25, 0.3) | -0.032(-0.049,-0.015) |
| Western Sub-Saharan Africa | 5343(4827.17,5899.64) | 4.23(3.84,4.65) | 13483.04(12177.74,14910.75) | 4.21(3.82,4.62) | 1.52(1.5, 1.54) | -0.024(-0.052,0.003) |

ASIR = age standardized incidence rate; EAPC = estimated annual percentage change; SDI = socio-demographic index; 95% UI = 95% uncertainty interval; 95% CI = 95% confidence interval.
