# Supplementary material for: Global, regional, and national burden of musculoskeletal disorders, 1990–2021: an analysis of the global burden of disease study 2021 and forecast to 2035
Source: Front Public Health. 2025 Aug 1;13:1562701. doi: 10.3389/fpubh.2025.1562701 (PMC12354483; doi:10.3389/fpubh.2025.1562701)
Supplement: Supplementary file 2 [file Table_2.doc]

**Table S2** Age standardized DALYs rate (ASDR) of musculoskeletal disorders in 1990 and 2021, and estimated annual percentage change (EAPC) from 1990 to 2021 at the global and regional level.

| Group | **1990** | | **2021** | | **1990-2021** | |
| --- | --- | --- | --- | --- | --- | --- |
|  | **DALYs cases,**  **×1000 (95% UI)** | **ASDRs**  **per 100 000**  **(95% UI)** | **DALYs cases,**  **×1000 (95% UI)** | **ASDRs**  **per 100 000**  **(95% UI)** | **Total percent change**  **(95% UI)** | **EAPC, %,**  **(95% CI)** |
| Global | 86285.11(63579.33,114732.38) | 1.89(1.38,2.52) | 161877.7(118018.72,216145.51) | 1.91(1.39,2.55) | 0.88(0.83, 0.93) | 0.088(0.065,0.111) |
| SDI |  |  |  |  |  |  |
| High | 22938.06(16777.75,30647.77) | 2.28(1.67,3.04) | 35358.16(26024.61,47354.4) | 2.36(1.72,3.12) | 0.54(0.5, 0.59) | 0.137(0.130,0.143) |
| High-middle | 19409.24(14212.96,26018.55) | 1.84(1.34,2.47) | 31547.67(22868.42,42534.62) | 1.83(1.33,2.45) | 0.63(0.58, 0.68) | 0.052(0.016,0.088) |
| Middle | 23616.18(17359.81,31379.35) | 1.74(1.27,2.32) | 49358.01(35999.36,66293.47) | 1.8(1.32,2.4) | 1.09(1.02, 1.17) | 0.182(0.155,0.209) |
| Low-middle | 14936.03(11104.83,19597.1) | 1.82(1.34,2.41) | 32760.96(24102.93,43251.19) | 1.91(1.41,2.53) | 1.19(1.15, 1.24) | 0.182(0.139,0.226) |
| Low | 5297.71(3924.88,6985.22) | 1.68(1.23,2.23) | 12721.88(9336.96,16826.31) | 1.71(1.26,2.29) | 1.4(1.37, 1.43) | 0.088(0.052,0.123) |
| Regions |  |  |  |  |  |  |
| Andean Latin America | 463.55(339.64,615.48) | 1.69(1.23,2.27) | 1166.34(849.01,1555.29) | 1.81(1.31,2.41) | 1.52(1.44, 1.6) | 0.238(0.224,0.253) |
| Australasia | 534.99(393.99,708.85) | 2.4(1.77,3.18) | 977.16(718.7,1311.01) | 2.42(1.78,3.22) | 0.83(0.75, 0.9) | 0.058(0.031,0.086) |
| Caribbean | 481.13(351,641.84) | 1.6(1.16,2.13) | 871.16(635.4,1165.68) | 1.68(1.23,2.24) | 0.81(0.76, 0.87) | 0.175(0.167,0.183) |
| Central Asia | 920.05(670.47,1240.31) | 1.68(1.22,2.28) | 1630.45(1186.08,2205.1) | 1.77(1.28,2.41) | 0.77(0.73, 0.82) | 0.206(0.187,0.225) |
| Central Europe | 2982.67(2198.56,4017.66) | 2.12(1.57,2.86) | 3525.75(2579.53,4810.32) | 2.16(1.58,2.92) | 0.18(0.15, 0.22) | 0.063(0.056,0.070) |
| Central Latin America | 2457.05(1825.22,3244.96) | 2.07(1.52,2.75) | 5818.71(4272.91,7735.43) | 2.21(1.63,2.94) | 1.37(1.29, 1.46) | 0.191(0.168,0.214) |
| Central Sub-Saharan Africa | 547.08(403.29,723.47) | 1.65(1.21,2.19) | 1436.26(1045.28,1912.02) | 1.66(1.21,2.2) | 1.63(1.55, 1.7) | 0.003(-0.012,0.017) |
| East Asia | 17290.99(12645.74,23025.6) | 1.62(1.17,2.16) | 31710.05(22689.41,43333.38) | 1.59(1.15,2.14) | 0.83(0.74, 0.94) | 0.089(0.032,0.145) |
| Eastern Europe | 5210.02(3823.36,7046.1) | 1.99(1.46,2.69) | 5824.09(4222.19,7866.68) | 2.02(1.48,2.75) | 0.12(0.09, 0.15) | 0.125(0.089,0.161) |
| Eastern Sub-Saharan Africa | 1725.18(1264.83,2281.43) | 1.57(1.15,2.1) | 4255.23(3087.7,5654.15) | 1.61(1.17,2.15) | 1.47(1.43, 1.5) | 0.087(0.068,0.105) |
| High-income Asia Pacific | 4875.22(3570.88,6536.74) | 2.46(1.8,3.29) | 7066.37(5136.2,9576.08) | 2.48(1.82,3.32) | 0.45(0.39, 0.52) | 0.111(0.070,0.152) |
| High-income North America | 8058.1(5909.23,10777.64) | 2.56(1.88,3.42) | 13206.75(9792.6,17399.9) | 2.76(2.03,3.6) | 0.64(0.56, 0.71) | 0.257(0.244,0.270) |
| North Africa and Middle East | 4530.9(3315.92,5953.54) | 1.89(1.38,2.51) | 11658.74(8451.25,15506.36) | 2.01(1.46,2.67) | 1.57(1.51, 1.65) | 0.221(0.208,0.235) |
| Oceania | 65.95(48.25,87.46) | 1.51(1.1,2.03) | 163.79(119.67,219.92) | 1.54(1.12,2.07) | 1.48(1.42, 1.54) | 0.076(0.066,0.085) |
| South Asia | 14585.62(10789.39,19115.47) | 1.86(1.38,2.45) | 33651.98(24769.72,44294.68) | 1.95(1.44,2.56) | 1.31(1.26, 1.37) | 0.187(0.104,0.271) |
| Southeast Asia | 5303.28(3909.49,6972.58) | 1.53(1.12,2.03) | 11833.03(8619.52,15888.08) | 1.62(1.19,2.17) | 1.23(1.16, 1.31) | 0.202(0.196,0.207) |
| Southern Latin America | 1185.37(880.33,1573.79) | 2.49(1.85,3.3) | 2004.13(1475.19,2665.8) | 2.56(1.89,3.41) | 0.69(0.64, 0.74) | 0.114(0.071,0.158) |
| Southern Sub-Saharan Africa | 597.15(440.42,788.38) | 1.68(1.23,2.23) | 1198.59(878.75,1581.68) | 1.7(1.25,2.26) | 1.01(0.97, 1.06) | 0.085(0.073,0.097) |
| Tropical Latin America | 2760.82(2045.81,3661.86) | 2.24(1.66,2.99) | 5780.3(4256.96,7696.95) | 2.26(1.67,3.01) | 1.09(1.02, 1.18) | -0.068(-0.128,-0.009) |
| Western Europe | 9749.74(7131.73,13076.95) | 2.05(1.5,2.74) | 12957.49(9378.7,17465.62) | 2.08(1.51,2.79) | 0.33(0.3, 0.36) | 0.049(0.031,0.067) |
| Western Sub-Saharan Africa | 1960.24(1433.69,2608.89) | 1.61(1.18,2.15) | 5141.32(3746.2,6816.79) | 1.68(1.22,2.24) | 1.62(1.59, 1.65) | 0.141(0.130,0.151) |

ASDR = age standardized deaths rate; EAPC = estimated annual percentage change; SDI = socio-demographic index; 95% UI = 95% uncertainty interval; 95% CI = 95% confidence interval.
