# Supplementary material for: Global, regional, and national burden of musculoskeletal disorders, 1990–2021: an analysis of the global burden of disease study 2021 and forecast to 2035
Source: Front Public Health. 2025 Aug 1;13:1562701. doi: 10.3389/fpubh.2025.1562701 (PMC12354483; doi:10.3389/fpubh.2025.1562701)
Supplement: Supplementary file 3 [file Table_3.doc]

**Table S3** Age standardized mortality rate (ASMR) of musculoskeletal disorders in 1990 and 2021, and estimated annual percent age change (EAPC) from 1990 to 2021 at the global and regional level.

| Group | **1990** | | **2021** | | **1990-2021** | |
| --- | --- | --- | --- | --- | --- | --- |
|  | **Deaths cases**  **(95%UI)** | **ASMRs**  **per100000**  **(95%UI)** | **Deaths cases**  **(95%UI)** | **ASMRs**  **per100000**  **(95%UI)** | **Total percent change**  **(95%UI)** | **EAPC, %,**  **(95%CI)** |
| Global | 58380.19(52986.95, 62644.45) | 1.55(1.41, 1.66) | 118499.5(103131.36, 128548.08) | 1.44(1.25, 1.56) | 1.03(0.84, 1.18) | -0.265(-0.361,-0.168) |
| SDI |  |  |  |  |  |  |
| High | 20519.6(19148.4, 21219.28) | 1.89(1.77, 1.96) | 31841.56(27547.74, 34271.5) | 1.44(1.27, 1.53) | 0.55(0.43, 0.63) | -1.040(-1.261,-0.819) |
| High-middle | 11449.38(10496.63, 12475.76) | 1.29(1.17, 1.4) | 20685.99(17913.57, 23227.25) | 1.13(0.98, 1.27) | 0.81(0.59, 1.02) | -0.548(-0.716,-0.380) |
| Middle | 14039.53(11985.04, 15695.46) | 1.34(1.13, 1.48) | 32463.73(27737.65, 36104.08) | 1.33(1.13, 1.48) | 1.31(0.98, 1.59) | 0.095(-0.022,0.213) |
| Low-middle | 9603.58(7606.2, 11152.5) | 1.76(1.4, 2.03) | 26747.35(20755.76, 29777.27) | 2.13(1.64, 2.38) | 1.79(1.39, 2.15) | 0.733(0.645,0.822) |
| Low | 2709.37(1990.75, 3556.93) | 1.28(0.95, 1.68) | 6677.1(4982.7, 8408.8) | 1.41(1.11, 1.75) | 1.46(1.12, 1.82) | 0.478(0.311,0.646) |
| Regions |  |  |  |  |  |  |
| Andean Latin America | 291.86(239.93, 330.36) | 1.42(1.14, 1.65) | 737.39(599.57, 887.57) | 1.25(1.02, 1.51) | 1.53(1, 2.13) | -0.566(-0.822,-0.309) |
| Australasia | 537.11(490.16, 571.4) | 2.33(2.13, 2.49) | 1157.92(985.79, 1287.51) | 1.98(1.71, 2.19) | 1.16(0.92, 1.42) | -0.455(-0.615,-0.296) |
| Caribbean | 487.34(438.7, 567.51) | 1.93(1.73, 2.19) | 1030.62(883.07, 1238.38) | 1.94(1.65, 2.35) | 1.11(0.85, 1.41) | -0.083(-0.204,0.039) |
| Central Asia | 40.62(34.68, 52.33) | 0.09(0.08, 0.12) | 438.47(388.99, 485.84) | 0.62(0.55, 0.68) | 9.79(7.29, 11.92) | 6.138(4.854,7.438) |
| Central Europe | 1633.3(1565.99, 1695.52) | 1.14(1.09, 1.19) | 1398.96(1270.19, 1515.21) | 0.65(0.59, 0.7) | -0.14(-0.22, -0.07) | -2.015(-2.301,-1.728) |
| Central Latin America | 2383.51(2304.24, 2459.13) | 2.62(2.5, 2.7) | 6191.48(5084.91, 6903.15) | 2.47(2.03, 2.75) | 1.6(1.09, 1.9) | -0.132(-0.228,-0.036) |
| Central Sub-Saharan Africa | 279.7(122.66, 440.97) | 1.09(0.46, 1.88) | 620.36(293.96, 1058.46) | 0.99(0.47, 1.74) | 1.22(0.63, 1.95) | -0.450(-0.518,-0.381) |
| East Asia | 10452.67(8713.32, 12660.73) | 1.22(1.01, 1.48) | 20465.63(16108.72, 25449.97) | 1.1(0.86, 1.35) | 0.96(0.39, 1.52) | -0.145(-0.438,0.148) |
| Eastern Europe | 2037.84(1940.08, 2102.3) | 0.77(0.74, 0.8) | 4832.45(4391.15, 5237.89) | 1.45(1.32, 1.57) | 1.37(1.15, 1.64) | 1.271(0.534,2.012) |
| Eastern Sub-Saharan Africa | 583.36(333.85, 1010.42) | 0.65(0.35, 1.24) | 1099.28(646.08, 2063.05) | 0.55(0.31, 1.04) | 0.88(0.45, 1.38) | -0.702(-0.777,-0.627) |
| High-income Asia Pacific | 3596.51(3337.42, 3742.88) | 1.89(1.73, 1.96) | 6613.17(5424.27, 7406.57) | 1.27(1.08, 1.39) | 0.84(0.59, 1.01) | -1.590(-1.870,-1.309) |
| High-income North America | 6870.33(6409.79, 7139.29) | 1.98(1.86, 2.05) | 9678.29(8485.35, 10350.91) | 1.52(1.35, 1.62) | 0.41(0.29, 0.47) | -1.142(-1.637,-0.645) |
| North Africa and Middle East | 1591.29(1335.68, 1910.43) | 0.83(0.67, 1) | 3741.29(2970.49, 4528.45) | 0.84(0.66, 1.01) | 1.35(0.87, 1.8) | 0.388(0.213,0.564) |
| Oceania | 16.09(6.11, 26.51) | 0.35(0.17, 0.56) | 38.84(14.18, 63.42) | 0.35(0.15, 0.55) | 1.41(0.87, 2.26) | -0.118(-0.203,-0.033) |
| South Asia | 10011.64(7785, 11652.77) | 2.23(1.75, 2.58) | 30896.17(24263.53, 34951.08) | 2.52(1.98, 2.86) | 2.09(1.63, 2.57) | 0.493(0.372,0.614) |
| Southeast Asia | 3067.35(2348.06, 3756.82) | 0.92(0.69, 1.09) | 6091.79(4702.71, 7038.29) | 0.98(0.74, 1.12) | 0.99(0.62, 1.33) | 0.081(-0.008,0.170) |
| Southern Latin America | 695.36(656.07, 736.4) | 1.56(1.47, 1.66) | 1112.21(1017.48, 1203.19) | 1.3(1.19, 1.4) | 0.6(0.46, 0.77) | -0.152(-0.373,0.071) |
| Southern Sub-Saharan Africa | 594.36(446.86, 690.14) | 1.95(1.46, 2.33) | 1192.7(990.08, 1377.64) | 2.09(1.73, 2.4) | 1.01(0.67, 1.48) | 0.072(-0.262,0.406) |
| Tropical Latin America | 1384.57(1335.28, 1437.83) | 1.27(1.21, 1.33) | 3624.59(3279.93, 3854.23) | 1.45(1.31, 1.54) | 1.62(1.36, 1.78) | 0.647(0.428,0.866) |
| Western Europe | 11126.45(10239.81, 11685.51) | 1.92(1.77, 2.02) | 15445.95(12961.09, 16792.71) | 1.41(1.22, 1.51) | 0.39(0.27, 0.48) | -1.009(-1.124,-0.894) |
| Western Sub-Saharan Africa | 698.9(492.22, 931.48) | 0.68(0.47, 0.91) | 2091.96(1194.39, 2668.63) | 0.83(0.54, 1.03) | 1.99(1.21, 2.69) | 0.704(0.666,0.742) |

ASMR=age standardized deaths rate;EAPC= estimated annual percentage change;SDI= socio-demographic index;95%UI=95% uncertainty interval;95%CI=95% confidence interval.
