# Supplementary material for: Global, regional, and national burden of musculoskeletal disorders, 1990–2021: an analysis of the global burden of disease study 2021 and forecast to 2035
Source: Front Public Health. 2025 Aug 1;13:1562701. doi: 10.3389/fpubh.2025.1562701 (PMC12354483; doi:10.3389/fpubh.2025.1562701)
Supplement: Supplementary file 4 [file Table_4.doc]

**Table S4** Age standardized prevalence, incidence, DALYs, and deaths rate of of musculoskeletal disorders in 1990 and 2021, and estimated annual percentage change (EAPC) from 1990 to 2021 by 204 countries and territories

| **Group** | **Age standardized rate in 2021 (per 100 000 population) (95% UI)** | | | | **EAPC in age standardized rate (%) from 1990 to 2021 (95% CI)** | | | |
| --- | --- | --- | --- | --- | --- | --- | --- | --- |
|  | **Prevalence** | **Incidence** | **DALYs** | **Deaths** | **Prevalence** | **Incidence** | **DALYs** | **Deaths** |
| Afghanistan | 17698.19(16233.35, 19141.94) | 4840.67(4349.02, 5338.34) | 1748.67(1227.69, 2326.64) | 1.5(0.97, 2.28) | 0.158(0.154, 0.163) | 0.029(0.021, 0.037) | 0.158(0.144, 0.172) | 1.138(1.035, 1.241) |
| Albania | 20334.75(18960.44, 21706.78) | 6189.19(5548.95, 6910.77) | 2103.58(1552.67, 2826.79) | 0.69(0.51, 0.91) | 0.204(0.198, 0.211) | 0.039(0.035, 0.043) | 0.184(0.175, 0.193) | -0.972(-1.206, -0.738) |
| Algeria | 20342.7(19133.38, 21580.46) | 4864.25(4396.15, 5357.71) | 1995.33(1449.52, 2650.12) | 0.69(0.49, 0.88) | 0.303(0.294, 0.312) | 0.034(0.023, 0.044) | 0.281(0.268, 0.294) | 1.716(1.503, 1.930) |
| American Samoa | 18896.28(17907.67, 20012.21) | 4030.02(3662.5, 4437.29) | 1708.34(1235.75, 2283.35) | 0.93(0.72, 1.15) | 0.097(0.075, 0.119) | 0.010(-0.007, 0.027) | 0.071(0.051, 0.092) | 1.096(0.701, 1.493) |
| Andorra | 20355.78(19173.61, 21742.81) | 5054.57(4577.34, 5575.52) | 1946.82(1408.12, 2629.03) | 0.13(0.09, 0.17) | 0.064(0.058, 0.070) | -0.029(-0.036, -0.022) | 0.023(0.014, 0.033) | -0.830(-1.008, -0.651) |
| Angola | 17864.25(16695.31, 19016.99) | 4388.05(3945.66, 4848.6) | 1681.24(1212.23, 2245.81) | 0.95(0.43, 1.83) | 0.090(0.078, 0.102) | -0.061(-0.083, -0.039) | 0.063(0.055, 0.071) | -0.247(-0.322, -0.172) |
| Antigua and Barbuda | 18634.54(17489.12, 19740.73) | 3756.82(3403.05, 4108.79) | 1725.35(1256.12, 2312.97) | 2.34(2.14, 2.55) | 0.174(0.168, 0.180) | 0.029(0.017, 0.042) | 0.175(0.167, 0.183) | 0.891(0.664, 1.118) |
| Argentina | 25109.85(23692.47, 26567.66) | 5264.7(4760.57, 5803.98) | 2556.35(1880.79, 3408.3) | 1.17(1.08, 1.27) | 0.209(0.190, 0.227) | 0.027(-0.002, 0.056) | 0.217(0.191, 0.243) | 0.369(0.108, 0.630) |
| Armenia | 18877.56(17680.4, 20121.15) | 5213.62(4693.07, 5799.61) | 1842.75(1339.03, 2499.67) | 0.42(0.35, 0.47) | 0.374(0.356, 0.391) | 0.088(0.078, 0.098) | 0.381(0.359, 0.403) | 9.954(8.034, 11.909) |
| Australia | 24435.75(23112.27, 25909.57) | 5779.25(5225.21, 6342.19) | 2468.36(1820.49, 3286.93) | 1.96(1.68, 2.18) | 0.109(0.078, 0.139) | -0.078(-0.102, -0.055) | 0.055(0.021, 0.090) | -0.458(-0.602, -0.313) |
| Austria | 20207.86(19040, 21490.22) | 4771.53(4317.1, 5229.65) | 1957.26(1415.52, 2618.5) | 0.97(0.84, 1.08) | 0.129(0.096, 0.163) | -0.038(-0.073, -0.004) | 0.095(0.043, 0.146) | -1.054(-1.313, -0.793) |
| Azerbaijan | 18485.54(17260.29, 19690.11) | 5014.48(4529.02, 5542.98) | 1774.22(1277.62, 2414.67) | 0.03(0.01, 0.05) | 0.241(0.205, 0.276) | 0.092(0.075, 0.110) | 0.233(0.201, 0.265) | 4.500(3.410, 5.602) |
| Bahamas | 18989.64(17904.22, 20106.34) | 3782.09(3423.33, 4156.56) | 1820.33(1338.58, 2406.55) | 3.7(2.98, 4.54) | 0.146(0.137, 0.155) | 0.020(0.012, 0.029) | 0.125(0.110, 0.139) | 0.168(-0.062, 0.398) |
| Bahrain | 20275.89(19156.68, 21499.57) | 4705.23(4258.45, 5186.12) | 1986.61(1443.93, 2647.1) | 1.99(1.32, 2.44) | 0.186(0.181, 0.190) | -0.019(-0.031, -0.007) | 0.201(0.186, 0.216) | 1.401(0.866, 1.940) |
| Bangladesh | 22639.39(21418.24, 24048.9) | 4545.43(4070.47, 5052.07) | 2311.77(1707.98, 3072.61) | 1.96(1.42, 2.67) | 0.248(0.223, 0.273) | -0.054(-0.091, -0.018) | 0.227(0.190, 0.264) | -0.550(-0.842, -0.257) |
| Barbados | 19056.02(17972.32, 20218.94) | 3804.2(3440.81, 4175.45) | 1812.14(1329.01, 2406.93) | 3.55(2.87, 4.39) | 0.148(0.140, 0.155) | 0.024(0.016, 0.031) | 0.097(0.074, 0.119) | -0.201(-0.472, 0.071) |
| Belarus | 19850.22(18563.78, 21079.31) | 5598.18(5036.38, 6155.16) | 1918.1(1378.39, 2635.08) | 0.06(0.05, 0.07) | 0.182(0.163, 0.200) | 0.041(0.032, 0.049) | 0.158(0.130, 0.187) | 0.207(-0.147, 0.563) |
| Belgium | 20693.38(19497.08, 21982.69) | 5182.36(4678.4, 5715.53) | 2018.71(1478.23, 2736.28) | 1.16(0.99, 1.28) | 0.083(0.068, 0.097) | -0.035(-0.046, -0.024) | 0.017(-0.007, 0.042) | -1.796(-2.235, -1.354) |
| Belize | 18346.43(17287.06, 19524.97) | 3820.21(3467.5, 4179.43) | 1702.57(1231.99, 2263.33) | 1.91(1.65, 2.19) | 0.239(0.230, 0.248) | 0.090(0.083, 0.096) | 0.242(0.231, 0.253) | 1.298(0.736, 1.863) |
| Benin | 17597.36(16588.55, 18639.5) | 4166.37(3769.73, 4581.77) | 1661.75(1203.13, 2220.98) | 0.82(0.5, 1.08) | 0.126(0.115, 0.137) | -0.057(-0.085, -0.030) | 0.085(0.068, 0.101) | 0.546(0.413, 0.680) |
| Bermuda | 19436.3(18358.81, 20568.61) | 3853.18(3491.71, 4239.99) | 1805.48(1318, 2426.88) | 1.91(1.59, 2.35) | 0.145(0.140, 0.150) | 0.002(-0.004, 0.008) | 0.109(0.103, 0.115) | -1.246(-1.618, -0.873) |
| Bhutan | 20878.54(19684.08, 22154.62) | 4447.02(3995.21, 4926.63) | 2108.34(1560.81, 2779.5) | 2.6(1.77, 3.7) | 0.279(0.271, 0.286) | 0.009(0.001, 0.017) | 0.269(0.257, 0.280) | 0.039(0.000, 0.078) |
| Bolivia (Plurinational State of) | 19198.25(18089.67, 20327.56) | 3811.35(3456.74, 4198.94) | 1809.28(1323.74, 2412.43) | 1.93(1.37, 2.61) | 0.241(0.233, 0.249) | 0.084(0.062, 0.107) | 0.223(0.204, 0.243) | -0.124(-0.163, -0.084) |
| Bosnia and Herzegovina | 20373.84(19021.55, 21772.75) | 6055.76(5468.64, 6707.05) | 2058.27(1506.09, 2762.43) | 0.59(0.43, 0.78) | 0.289(0.276, 0.302) | 0.092(0.084, 0.100) | 0.264(0.251, 0.277) | -1.181(-1.399, -0.963) |
| Botswana | 17777.85(16794.2, 18735.2) | 3983.08(3582.76, 4368.25) | 1636.55(1190.04, 2177) | 1.22(0.81, 1.72) | 0.220(0.214, 0.226) | 0.053(0.045, 0.061) | 0.141(0.125, 0.157) | -0.374(-0.630, -0.117) |
| Brazil | 22660.51(21373.23, 24015.98) | 5150.96(4649.49, 5682.03) | 2267.32(1670.42, 3018.19) | 1.43(1.3, 1.52) | -0.050(-0.102, 0.003) | 0.061(0.035, 0.088) | -0.074(-0.135, -0.014) | 0.598(0.378, 0.817) |
| Brunei Darussalam | 23040.69(21815.22, 24450.09) | 4937.13(4467.22, 5435.28) | 2238(1631.99, 2990.81) | 1.94(1.54, 2.37) | 0.234(0.209, 0.258) | -0.043(-0.060, -0.026) | 0.221(0.191, 0.251) | -0.586(-0.776, -0.395) |
| Bulgaria | 20617.28(19275.75, 22001.89) | 6100.27(5470.13, 6742.97) | 2058.31(1499.35, 2823.62) | 0.27(0.23, 0.31) | 0.108(0.104, 0.113) | -0.014(-0.019, -0.010) | 0.090(0.083, 0.098) | -1.326(-1.711, -0.939) |
| Burkina Faso | 16785.06(15749.16, 17822.51) | 4115.49(3708.91, 4536.64) | 1618.24(1180.76, 2167.57) | 0.92(0.56, 1.23) | 0.061(0.050, 0.073) | -0.084(-0.109, -0.059) | 0.065(0.050, 0.081) | 0.447(0.349, 0.544) |
| Burundi | 15279.79(14142.86, 16329) | 4141.96(3731.13, 4560.76) | 1449.73(1044.8, 1963.89) | 0.51(0.24, 1.15) | -0.254(-0.325, -0.184) | -0.146(-0.156, -0.136) | -0.344(-0.431, -0.256) | -1.485(-1.666, -1.303) |
| Cabo Verde | 17453.74(16496.69, 18504.04) | 3984.36(3596.4, 4364.91) | 1625.01(1175.35, 2175.06) | 0.57(0.41, 0.73) | 0.132(0.122, 0.142) | -0.113(-0.140, -0.086) | 0.028(0.008, 0.048) | 0.278(0.172, 0.383) |
| Cambodia | 16336.64(15255.08, 17480.36) | 3594.46(3252.26, 3953.52) | 1564.81(1145.62, 2073.9) | 1.42(0.71, 1.99) | 0.142(0.133, 0.151) | -0.122(-0.130, -0.114) | 0.070(0.067, 0.073) | -0.014(-0.058, 0.030) |
| Cameroon | 17963.26(16870.11, 18983.32) | 4245.34(3853.77, 4690.1) | 1712(1238.84, 2279.36) | 1.02(0.59, 1.4) | 0.073(0.067, 0.078) | -0.094(-0.118, -0.070) | 0.041(0.033, 0.049) | 0.061(-0.050, 0.172) |
| Canada | 25037.69(23600.58, 26519) | 4872.92(4400.94, 5394.72) | 2511.65(1853.02, 3314.88) | 1.59(1.4, 1.75) | 0.141(0.130, 0.152) | -0.104(-0.131, -0.077) | 0.079(0.061, 0.097) | -0.909(-1.331, -0.485) |
| Central African Republic | 16778.24(15721.2, 17798.43) | 4324(3894.65, 4756.02) | 1611.18(1180.01, 2145.92) | 1.1(0.48, 1.91) | -0.021(-0.029, -0.012) | -0.042(-0.053, -0.030) | -0.022(-0.032, -0.013) | -0.571(-0.647, -0.495) |
| Chad | 16957.66(15884.46, 18010) | 4296.45(3882.72, 4743) | 1641.19(1198.72, 2173.77) | 0.69(0.45, 1.01) | -0.034(-0.060, -0.009) | -0.152(-0.205, -0.099) | -0.076(-0.113, -0.039) | 0.585(0.436, 0.734) |
| Chile | 25281.83(23883.97, 26775.87) | 5330.34(4843.41, 5865.88) | 2572.9(1900.69, 3380.99) | 1.47(1.32, 1.62) | -0.098(-0.177, -0.019) | 0.027(-0.000, 0.054) | -0.195(-0.292, -0.098) | -1.505(-1.756, -1.252) |
| China | 17403.52(16414.43, 18413.98) | 3629.61(3307.36, 3954.47) | 1578.37(1140.06, 2129.43) | 1.08(0.84, 1.35) | 0.213(0.161, 0.265) | -0.183(-0.262, -0.104) | 0.080(0.021, 0.138) | -0.197(-0.502, 0.109) |
| Colombia | 22215.63(20900.06, 23511.33) | 4509.32(4088.95, 4977.63) | 2247.58(1658.87, 2997.55) | 2.27(1.87, 2.71) | 0.271(0.262, 0.279) | 0.067(0.055, 0.078) | 0.323(0.306, 0.340) | 0.664(0.386, 0.944) |
| Comoros | 16789(15813.49, 17738.78) | 4069.72(3666.04, 4481.29) | 1589.69(1154.4, 2129.78) | 0.61(0.34, 1.21) | 0.157(0.150, 0.163) | 0.001(-0.015, 0.017) | 0.135(0.123, 0.147) | -0.567(-0.748, -0.385) |
| Congo | 17852.36(16827.24, 18913.8) | 4270.21(3867.15, 4707.77) | 1686.59(1227.72, 2253.12) | 1.28(0.64, 1.95) | 0.090(0.060, 0.120) | -0.005(-0.016, 0.006) | 0.045(0.011, 0.079) | -0.894(-1.046, -0.741) |
| Cook Islands | 19187.28(18191.29, 20269.73) | 4077.78(3712.21, 4484.89) | 1717.76(1238.31, 2318.82) | 0.01(0, 0.01) | 0.227(0.216, 0.239) | 0.134(0.123, 0.145) | 0.211(0.203, 0.219) | -0.017(-0.391, 0.358) |
| Costa Rica | 22070.93(20858.37, 23331.04) | 4341.24(3942.63, 4786.33) | 2199.46(1619.55, 2909.8) | 2.34(2.01, 2.64) | 0.179(0.176, 0.183) | 0.009(0.006, 0.011) | 0.175(0.169, 0.182) | 0.438(0.291, 0.584) |
| Croatia | 17522.93(16477.59, 18535.42) | 4187.8(3776.55, 4600.48) | 1664.1(1205.88, 2227.49) | 0.75(0.44, 1.02) | 0.147(0.121, 0.172) | 0.006(-0.026, 0.037) | 0.136(0.098, 0.173) | -1.048(-1.437, -0.657) |
| Cuba | 20478.64(19140.34, 21835.52) | 6011.49(5388.87, 6669.39) | 2061.08(1512.66, 2772.32) | 0.89(0.78, 1.01) | 0.288(0.263, 0.313) | 0.007(-0.014, 0.029) | 0.275(0.240, 0.309) | 0.179(-0.123, 0.482) |
| Cyprus | 18377.99(17367.99, 19384.03) | 3670.1(3365.28, 3989.56) | 1693.53(1231.45, 2281.02) | 1.65(1.41, 1.88) | 0.185(0.173, 0.197) | -0.009(-0.021, 0.003) | 0.119(0.109, 0.129) | -2.529(-2.767, -2.290) |
| Czechia | 20766.74(19555.68, 22094.15) | 5148.55(4629.73, 5694.34) | 2074.1(1521.92, 2763.29) | 2.08(1.67, 2.53) | 0.072(0.066, 0.078) | -0.047(-0.051, -0.042) | 0.059(0.050, 0.067) | -0.903(-1.452, -0.352) |
| Côte d'Ivoire | 21608.49(20153.96, 23129.38) | 6341.32(5686.87, 7058.82) | 2187.66(1598.21, 2979.83) | 0.5(0.44, 0.56) | 0.090(0.079, 0.100) | -0.057(-0.088, -0.025) | 0.108(0.086, 0.130) | 0.770(0.624, 0.917) |
| Democratic People's Republic of Korea | 18023.56(16934.11, 19058.79) | 4050.54(3672.68, 4448.76) | 1743.85(1261.54, 2346.22) | 1.52(1.09, 1.99) | 0.054(0.050, 0.058) | -0.131(-0.141, -0.120) | 0.018(0.011, 0.026) | 0.336(0.163, 0.509) |
| Democratic Republic of the Congo | 17167.97(16145.19, 18245.51) | 4327.77(3910.29, 4768.1) | 1645.37(1206.57, 2185.77) | 0.97(0.42, 1.75) | -0.045(-0.068, -0.021) | -0.095(-0.109, -0.081) | -0.028(-0.045, -0.011) | -0.423(-0.506, -0.340) |
| Denmark | 22937.27(21431.18, 24525.75) | 5093.16(4481.36, 5814.56) | 2385.58(1732.93, 3200.78) | 1.82(1.56, 2.04) | -0.169(-0.215, -0.123) | -0.352(-0.412, -0.292) | -0.265(-0.334, -0.196) | -0.074(-0.527, 0.381) |
| Djibouti | 16597.26(15623.87, 17573.07) | 3948.09(3561.91, 4334.41) | 1539.99(1118.78, 2072.88) | 0.51(0.25, 1.04) | 0.168(0.146, 0.190) | -0.070(-0.079, -0.061) | 0.075(0.056, 0.093) | 0.060(-0.101, 0.222) |
| Dominica | 18590.38(17575.87, 19739) | 3770.14(3401.25, 4137.92) | 1720.12(1245.93, 2296.55) | 1.65(1.3, 2.06) | 0.160(0.150, 0.169) | -0.039(-0.050, -0.029) | 0.117(0.107, 0.126) | 0.115(0.031, 0.200) |
| Dominican Republic | 18322.79(17258.64, 19395.74) | 3811.73(3441.05, 4193.44) | 1680.34(1217.95, 2258.61) | 1.58(1.15, 2.05) | 0.228(0.214, 0.241) | 0.098(0.083, 0.114) | 0.218(0.197, 0.239) | 0.307(-0.012, 0.626) |
| Ecuador | 19076.63(18050.13, 20203.53) | 3615.95(3307.85, 3950.61) | 1761.1(1277.92, 2362.75) | 1.72(1.38, 2.08) | 0.159(0.123, 0.194) | -0.027(-0.086, 0.032) | 0.133(0.087, 0.179) | 0.540(0.055, 1.028) |
| Egypt | 20482.97(19264.71, 21761.17) | 4952.38(4479.97, 5460.32) | 2017.58(1472.67, 2678.35) | 0.4(0.31, 0.49) | 0.288(0.270, 0.307) | 0.098(0.075, 0.121) | 0.307(0.280, 0.334) | 0.853(0.619, 1.087) |
| El Salvador | 21296.08(20028.76, 22660) | 4397.07(3959.71, 4868.13) | 2073.15(1514.76, 2778.15) | 1.23(0.95, 1.53) | 0.319(0.310, 0.328) | 0.120(0.110, 0.131) | 0.355(0.342, 0.367) | 0.514(0.398, 0.630) |
| Equatorial Guinea | 18558.6(17489.96, 19649.2) | 4330.56(3914.86, 4757.64) | 1754.53(1271.88, 2318.65) | 1.18(0.53, 1.87) | 0.386(0.361, 0.412) | -0.019(-0.034, -0.003) | 0.306(0.278, 0.334) | -0.042(-0.172, 0.089) |
| Eritrea | 15036.01(13858.07, 16129.39) | 3915.41(3524.78, 4293.32) | 1394.16(992.98, 1883.81) | 0.62(0.29, 1.27) | 0.315(0.306, 0.325) | 0.053(0.044, 0.062) | 0.359(0.350, 0.369) | -0.120(-0.195, -0.045) |
| Estonia | 20289.38(19128.03, 21540.7) | 5585.77(5025.38, 6171.27) | 2024.7(1476.4, 2749.89) | 1.95(1.69, 2.25) | 0.246(0.233, 0.259) | 0.048(0.035, 0.061) | 0.267(0.238, 0.297) | 0.241(-0.685, 1.176) |
| Eswatini | 17145.02(16170.79, 18091.03) | 3762.23(3414.12, 4121.62) | 1577.44(1166.63, 2090.86) | 2.29(1.37, 3.51) | 0.165(0.150, 0.180) | -0.012(-0.032, 0.007) | 0.079(0.053, 0.105) | 0.451(-0.121, 1.026) |
| Ethiopia | 17243.95(16199.48, 18286.67) | 4322.58(3905.65, 4751.35) | 1612.68(1171.19, 2155.87) | 0.47(0.26, 0.93) | 0.190(0.149, 0.231) | -0.109(-0.134, -0.083) | 0.091(0.042, 0.140) | -2.195(-2.398, -1.992) |
| Fiji | 18422.25(17417.31, 19486.26) | 3920.86(3550.93, 4290.53) | 1647.87(1178.55, 2222.01) | 0.16(0.11, 0.22) | 0.169(0.160, 0.179) | 0.004(-0.001, 0.010) | 0.107(0.102, 0.111) | -0.173(-0.373, 0.028) |
| Finland | 19450.25(18382.79, 20606.99) | 4743.51(4287.76, 5182.69) | 1866.44(1368.98, 2497.19) | 1.44(1.24, 1.6) | 0.153(0.118, 0.187) | -0.036(-0.052, -0.020) | 0.092(0.038, 0.147) | -2.528(-2.637, -2.419) |
| France | 20195.76(19001.11, 21442.93) | 5096.1(4606.76, 5594.23) | 1963.76(1429.71, 2619.8) | 1.85(1.58, 2.05) | 0.132(0.113, 0.151) | -0.007(-0.028, 0.013) | 0.123(0.088, 0.157) | 0.088(-0.526, 0.706) |
| Gabon | 18283.3(17272.88, 19305.26) | 4274.83(3866.9, 4717.57) | 1722.66(1250.92, 2303.9) | 1.37(0.67, 2.15) | 0.231(0.225, 0.236) | 0.050(0.044, 0.056) | 0.190(0.183, 0.198) | -0.208(-0.423, 0.007) |
| Gambia | 17327.36(16279.76, 18354.9) | 4009.62(3639.47, 4383.35) | 1613.74(1171.74, 2170.7) | 0.88(0.6, 1.17) | 0.175(0.160, 0.189) | -0.013(-0.046, 0.019) | 0.130(0.110, 0.150) | 1.102(0.953, 1.252) |
| Georgia | 17180.8(16069.76, 18330.4) | 4762.87(4288.52, 5277.1) | 1621.02(1171.41, 2193.29) | 0.34(0.29, 0.39) | 0.024(-0.013, 0.060) | -0.058(-0.089, -0.027) | -0.027(-0.063, 0.008) | 9.974(7.947, 12.038) |
| Germany | 22274.6(21059.55, 23703.18) | 5601.62(5050.48, 6196.42) | 2249.19(1641.61, 2991.85) | 1.12(0.96, 1.24) | 0.066(0.052, 0.081) | -0.063(-0.078, -0.047) | 0.041(0.022, 0.060) | -0.069(-0.668, 0.533) |
| Ghana | 17707.46(16727.18, 18726.1) | 3952.26(3612.59, 4300.57) | 1648.39(1193.72, 2221.47) | 1.09(0.78, 1.45) | 0.091(0.061, 0.120) | -0.092(-0.105, -0.079) | 0.092(0.079, 0.106) | 2.129(1.747, 2.513) |
| Greece | 20800.78(19639.55, 22124.37) | 5063.15(4582.7, 5603.06) | 2049.25(1487.09, 2747.17) | 0.7(0.62, 0.76) | 0.253(0.192, 0.314) | 0.015(-0.014, 0.044) | 0.237(0.187, 0.287) | 0.927(0.339, 1.519) |
| Greenland | 24263.14(22803.77, 25806.27) | 4794.53(4315.88, 5316.06) | 2387.62(1769.94, 3148.77) | 1.49(1.11, 1.85) | 0.258(0.247, 0.269) | -0.040(-0.066, -0.013) | 0.235(0.219, 0.252) | -1.306(-1.404, -1.207) |
| Grenada | 18317.04(17257.77, 19439.42) | 3753.08(3408.5, 4114.35) | 1704.97(1240.48, 2279.83) | 2.5(2.18, 2.82) | 0.224(0.209, 0.240) | 0.025(0.013, 0.037) | 0.177(0.157, 0.196) | 0.425(0.217, 0.633) |
| Guam | 18932.93(17884.93, 20008.44) | 3965.81(3609.9, 4348.71) | 1737.49(1255, 2349.94) | 0.55(0.46, 0.64) | 0.173(0.157, 0.188) | 0.051(0.040, 0.062) | 0.160(0.138, 0.182) | -0.315(-0.846, 0.220) |
| Guatemala | 21328.16(19967.01, 22657.34) | 4580.92(4127.61, 5065.14) | 2105.45(1534.7, 2808.68) | 1.4(1.2, 1.6) | 0.191(0.173, 0.209) | -0.020(-0.046, 0.006) | 0.180(0.152, 0.208) | 0.642(0.234, 1.052) |
| Guinea | 17099.87(16062.44, 18168) | 4175.2(3769.76, 4604.41) | 1643.08(1201.7, 2194.98) | 0.77(0.48, 1.09) | 0.075(0.064, 0.086) | -0.044(-0.073, -0.014) | 0.061(0.042, 0.081) | 0.504(0.374, 0.634) |
| Guinea-Bissau | 16895.97(15919.61, 17986.48) | 4060.48(3671.46, 4465.37) | 1615.26(1171.78, 2157.4) | 1.15(0.77, 1.57) | 0.093(0.088, 0.097) | -0.046(-0.068, -0.023) | 0.079(0.067, 0.091) | 0.334(0.208, 0.460) |
| Guyana | 18075.01(16976.69, 19135.77) | 3753.7(3386.07, 4116.41) | 1627.87(1190.78, 2156.55) | 1.2(0.93, 1.51) | 0.210(0.207, 0.213) | 0.041(0.039, 0.042) | 0.192(0.182, 0.203) | 0.573(0.125, 1.022) |
| Haiti | 16489.55(15415.6, 17584.67) | 3676.39(3316.94, 4038.7) | 1562(1145.65, 2053.36) | 2.62(1.54, 4.33) | 0.078(0.049, 0.107) | 0.012(-0.000, 0.023) | 0.020(-0.022, 0.063) | -0.242(-0.321, -0.163) |
| Honduras | 21217.9(19979.9, 22520.48) | 4367.54(3935.05, 4824.41) | 2145.52(1590.56, 2829.75) | 3.46(2.38, 4.73) | 0.234(0.230, 0.239) | 0.082(0.076, 0.088) | 0.248(0.239, 0.258) | 0.940(0.730, 1.151) |
| Hungary | 22308.31(20934.38, 23766.85) | 6517.02(5871.34, 7216.37) | 2308.38(1685.43, 3101.07) | 1.13(0.95, 1.32) | 0.104(0.097, 0.111) | -0.027(-0.031, -0.023) | 0.109(0.099, 0.119) | -1.497(-1.829, -1.165) |
| Iceland | 21394.01(20202.37, 22639.08) | 5225.32(4728.56, 5778.24) | 2113.98(1532.01, 2854.14) | 1.11(0.93, 1.26) | 0.021(0.009, 0.033) | -0.140(-0.150, -0.130) | -0.017(-0.029, -0.005) | -0.549(-0.700, -0.397) |
| India | 19948.28(18827.11, 21238.05) | 3796.17(3435.7, 4165.15) | 1905.75(1406.88, 2509.99) | 2.52(1.97, 2.88) | 0.194(0.126, 0.263) | -0.310(-0.442, -0.179) | 0.150(0.049, 0.251) | 0.675(0.493, 0.858) |
| Indonesia | 17698.77(16758.07, 18770.71) | 3788.63(3438.91, 4152.79) | 1666.86(1214.31, 2238.19) | 0.45(0.21, 0.56) | 0.261(0.252, 0.270) | 0.036(0.021, 0.050) | 0.237(0.227, 0.248) | 0.675(0.530, 0.820) |
| Iran (Islamic Republic of) | 21020.76(19740.11, 22354.05) | 5402.43(4878.71, 5935.39) | 2112.06(1540.29, 2812.31) | 0.73(0.46, 0.83) | 0.202(0.156, 0.248) | -0.110(-0.154, -0.066) | 0.183(0.135, 0.232) | 1.473(1.190, 1.757) |
| Iraq | 19218.78(17873.25, 20560.1) | 4838.77(4377.19, 5322.85) | 1849.48(1331.14, 2464.19) | 0.57(0.42, 0.73) | 0.208(0.193, 0.222) | -0.004(-0.008, 0.001) | 0.194(0.183, 0.205) | -0.826(-0.914, -0.738) |
| Ireland | 21455.73(20325.49, 22696.58) | 5284.69(4789.13, 5827.77) | 2164.15(1591.1, 2889.4) | 1.8(1.51, 2.01) | 0.112(0.102, 0.123) | -0.038(-0.055, -0.020) | 0.067(0.053, 0.081) | -1.151(-1.314, -0.989) |
| Israel | 21196.35(19973.41, 22467.43) | 5234.37(4682.75, 5790.36) | 2106.12(1546.12, 2821.73) | 0.95(0.8, 1.05) | -0.048(-0.095, 0.000) | -0.105(-0.132, -0.077) | -0.047(-0.082, -0.012) | -0.990(-1.201, -0.778) |
| Italy | 21361.65(20112.92, 22697.9) | 5388.27(4857.59, 5937.96) | 2130.53(1547.41, 2856.22) | 1.1(0.96, 1.2) | 0.144(0.129, 0.159) | -0.044(-0.055, -0.033) | 0.150(0.141, 0.159) | -0.252(-0.561, 0.057) |
| Jamaica | 18565.46(17496.14, 19689.5) | 3825.61(3448.8, 4201.16) | 1782.17(1312.43, 2359.5) | 3.05(2.33, 3.92) | 0.202(0.190, 0.213) | 0.029(0.013, 0.044) | 0.134(0.083, 0.186) | -0.456(-1.041, 0.132) |
| Japan | 25014.49(23656.42, 26455.05) | 5783.39(5231.48, 6375.02) | 2575.22(1887.56, 3443.43) | 1.29(1.12, 1.4) | 0.167(0.112, 0.223) | -0.090(-0.130, -0.050) | 0.124(0.074, 0.173) | -1.372(-1.751, -0.992) |
| Jordan | 20825.51(19618.66, 22031.76) | 4901.05(4428.8, 5383.65) | 2047.9(1487.32, 2717.32) | 0.99(0.76, 1.24) | 0.242(0.231, 0.253) | 0.027(0.018, 0.036) | 0.186(0.171, 0.201) | -1.045(-1.304, -0.786) |
| Kazakhstan | 18932.3(17768.65, 20126.26) | 5129.89(4639.85, 5675.28) | 1808.53(1310.04, 2433.98) | 0.76(0.67, 0.86) | 0.235(0.205, 0.265) | 0.041(0.021, 0.060) | 0.207(0.184, 0.230) | 9.787(5.910, 13.806) |
| Kenya | 18330.37(17318.44, 19392.43) | 4583.86(4140.87, 5054.67) | 1750.88(1273.77, 2341.02) | 0.57(0.35, 1.01) | 0.154(0.146, 0.162) | -0.008(-0.021, 0.004) | 0.124(0.117, 0.131) | 1.012(0.901, 1.123) |
| Kiribati | 18271.59(17295.14, 19330.25) | 4047.19(3653.49, 4436.61) | 1657.93(1203.5, 2225.04) | 0.07(0.04, 0.1) | 0.166(0.151, 0.180) | 0.123(0.103, 0.142) | 0.200(0.185, 0.214) | -0.151(-0.259, -0.043) |
| Kuwait | 21227(20041.6, 22461.04) | 4883.45(4424.07, 5369.87) | 2070.51(1498.16, 2747.58) | 0.51(0.42, 0.59) | 0.328(0.296, 0.361) | 0.193(0.163, 0.223) | 0.367(0.325, 0.409) | 4.233(2.975, 5.506) |
| Kyrgyzstan | 18360.68(17129.34, 19569.49) | 4994.87(4492.33, 5552.46) | 1824.96(1329.1, 2476.32) | 1.91(1.55, 2.3) | 0.179(0.153, 0.205) | -0.022(-0.031, -0.014) | 0.238(0.193, 0.284) | 4.403(3.352, 5.464) |
| Lao People's Democratic Republic | 16525.61(15551.18, 17523) | 3480.89(3143.07, 3838.11) | 1579.7(1164.62, 2099.77) | 1.33(0.65, 1.85) | 0.235(0.217, 0.254) | -0.059(-0.075, -0.043) | 0.187(0.174, 0.200) | -0.173(-0.262, -0.083) |
| Latvia | 19907.28(18678.15, 21152.9) | 5520.96(4971.07, 6119.67) | 1961.93(1431.17, 2659.48) | 1.53(1.34, 1.75) | 0.232(0.219, 0.244) | 0.017(0.010, 0.024) | 0.247(0.222, 0.271) | 0.494(-0.403, 1.399) |
| Lebanon | 20422.24(19150.04, 21778.27) | 4866.13(4402.14, 5358.89) | 1985.36(1431.05, 2647.24) | 0.63(0.5, 0.78) | 0.398(0.386, 0.410) | 0.089(0.077, 0.100) | 0.388(0.373, 0.403) | -1.462(-1.560, -1.364) |
| Lesotho | 16985.68(16017.73, 17942.39) | 3911.26(3532.94, 4283.75) | 1577.14(1156.11, 2081.03) | 2.08(1.38, 2.8) | 0.112(0.103, 0.120) | -0.073(-0.087, -0.060) | 0.012(0.000, 0.023) | 2.348(1.869, 2.828) |
| Liberia | 17059.9(16001.24, 18137.92) | 4077.07(3684.8, 4514.04) | 1578.55(1151.68, 2119.26) | 0.93(0.54, 1.23) | 0.108(0.087, 0.129) | -0.047(-0.056, -0.037) | 0.074(0.052, 0.095) | 0.622(0.465, 0.779) |
| Libya | 20244.64(19047.41, 21549.13) | 4873.82(4395.17, 5376.99) | 1972.19(1428.62, 2623.05) | 0.85(0.57, 1.14) | 0.227(0.196, 0.258) | 0.040(0.030, 0.049) | 0.219(0.182, 0.256) | 2.714(2.408, 3.021) |
| Lithuania | 20029.5(18843.11, 21202.16) | 5576.44(5042.05, 6146.37) | 1990.79(1457.7, 2689.18) | 1.96(1.7, 2.25) | 0.195(0.187, 0.203) | -0.001(-0.009, 0.006) | 0.186(0.158, 0.215) | 1.128(0.340, 1.922) |
| Luxembourg | 20862.91(19729.28, 22141.32) | 5163.96(4688.52, 5707.71) | 2048.1(1498.5, 2732.12) | 0.87(0.75, 0.97) | 0.117(0.100, 0.134) | -0.065(-0.073, -0.057) | 0.099(0.079, 0.119) | -1.210(-1.391, -1.027) |
| Madagascar | 16532.82(15572.69, 17527.09) | 4139.41(3728.11, 4571.15) | 1592.28(1169.98, 2129.54) | 0.45(0.23, 0.9) | -0.003(-0.009, 0.002) | -0.157(-0.175, -0.139) | -0.033(-0.042, -0.025) | -0.341(-0.408, -0.273) |
| Malawi | 16925.41(15886.04, 17962.64) | 4199.55(3775.93, 4649.08) | 1618.96(1181.31, 2164.97) | 0.68(0.36, 1.32) | 0.131(0.126, 0.136) | -0.017(-0.028, -0.007) | 0.130(0.122, 0.137) | -0.201(-0.379, -0.022) |
| Malaysia | 17626.21(16622.88, 18740.4) | 3454.2(3136.76, 3782.47) | 1626.78(1172.6, 2166.28) | 1.24(1.05, 1.44) | 0.215(0.201, 0.229) | -0.034(-0.060, -0.008) | 0.159(0.142, 0.177) | 0.191(0.070, 0.311) |
| Maldives | 16793.21(15827.94, 17914.19) | 3261.54(2961.29, 3581.15) | 1512.13(1078.97, 2042.6) | 0.53(0.41, 0.65) | 0.261(0.229, 0.292) | -0.041(-0.069, -0.014) | 0.170(0.133, 0.207) | -2.431(-2.519, -2.342) |
| Mali | 16245.51(15259.77, 17262.86) | 3904.37(3531.62, 4279.37) | 1522.68(1101.93, 2027.46) | 0.91(0.58, 1.23) | 0.135(0.123, 0.148) | 0.057(0.043, 0.071) | 0.135(0.120, 0.151) | 0.126(0.090, 0.162) |
| Malta | 21223.57(20060.04, 22540.61) | 5321.91(4827.92, 5869.77) | 2113.96(1560.16, 2848.67) | 0.97(0.82, 1.09) | 0.064(0.049, 0.079) | -0.048(-0.065, -0.031) | 0.033(0.012, 0.054) | -1.178(-1.354, -1.002) |
| Marshall Islands | 17672.2(16673.73, 18708.42) | 3822.88(3483.79, 4203.68) | 1580.34(1151.19, 2116.47) | 0.48(0.2, 0.76) | 0.133(0.126, 0.141) | -0.007(-0.012, -0.003) | 0.072(0.067, 0.078) | 0.498(0.316, 0.680) |
| Mauritania | 17398.86(16376.32, 18447.03) | 3959.16(3599.7, 4336.99) | 1627.25(1182.76, 2183.14) | 0.8(0.5, 1.11) | 0.176(0.164, 0.189) | 0.002(-0.018, 0.021) | 0.154(0.137, 0.170) | -0.392(-0.463, -0.321) |
| Mauritius | 18060.12(17030.66, 19190.34) | 3561.33(3220.16, 3926.43) | 1704.71(1254.31, 2261.57) | 1.96(1.77, 2.1) | 0.178(0.165, 0.192) | -0.023(-0.049, 0.002) | 0.217(0.200, 0.234) | 4.404(3.676, 5.137) |
| Mexico | 22585.91(21331.03, 23894.53) | 4400.67(3982.48, 4836.62) | 2251.37(1657.62, 2989.7) | 2.75(2.15, 3.14) | 0.152(0.116, 0.188) | 0.120(0.052, 0.188) | 0.141(0.091, 0.192) | -0.568(-0.721, -0.416) |
| Micronesia (Federated States of) | 18192.55(17164.35, 19334.98) | 4023.79(3658.33, 4441.28) | 1659.89(1202.05, 2231.86) | 0.55(0.22, 0.79) | 0.172(0.151, 0.192) | 0.070(0.051, 0.088) | 0.137(0.116, 0.157) | -0.074(-0.185, 0.037) |
| Monaco | 21071.49(19906.51, 22384.88) | 5081.53(4554.02, 5580.45) | 2056.54(1490.33, 2780.16) | 0.46(0.33, 0.58) | 0.024(0.020, 0.029) | -0.058(-0.066, -0.051) | -0.011(-0.018, -0.004) | 0.382(0.216, 0.548) |
| Mongolia | 18095.33(16848.24, 19334.42) | 4964.94(4469.3, 5471.58) | 1738.9(1265.95, 2334.22) | 1.6(1.11, 2.19) | 0.233(0.219, 0.247) | 0.004(-0.002, 0.010) | 0.191(0.182, 0.200) | 4.675(4.212, 5.139) |
| Montenegro | 21022.13(19610.66, 22472.08) | 6185.52(5575.29, 6870.51) | 2117.99(1545.46, 2853.05) | 0.38(0.28, 0.48) | 0.160(0.146, 0.173) | 0.041(0.034, 0.048) | 0.159(0.141, 0.177) | 0.323(0.170, 0.477) |
| Morocco | 20852.76(19594.95, 22145.3) | 5203.16(4674.25, 5747.29) | 2092.31(1520.21, 2760.71) | 1.05(0.5, 2.02) | 0.211(0.180, 0.243) | 0.021(-0.034, 0.075) | 0.212(0.157, 0.268) | 2.276(2.056, 2.495) |
| Mozambique | 16759.61(15687.57, 17870.41) | 4318.09(3869.39, 4773.24) | 1594.19(1161.25, 2139.41) | 0.72(0.33, 1.64) | 0.149(0.132, 0.167) | 0.006(0.000, 0.011) | 0.149(0.126, 0.171) | 0.710(0.568, 0.852) |
| Myanmar | 16048.52(15092.32, 17083.54) | 3248.67(2954.91, 3562.54) | 1483.12(1076.73, 1970.22) | 1.26(0.71, 1.64) | 0.335(0.322, 0.349) | 0.064(0.045, 0.082) | 0.245(0.229, 0.260) | -0.720(-0.935, -0.503) |
| Namibia | 17381.31(16362.82, 18354.01) | 4008.47(3632.32, 4363.16) | 1641.25(1201.28, 2181.31) | 1.39(0.88, 2.05) | 0.213(0.199, 0.227) | 0.047(0.038, 0.056) | 0.178(0.166, 0.190) | -0.271(-0.571, 0.031) |
| Nauru | 18414.19(17343.97, 19493.77) | 4057.43(3693.96, 4430.8) | 1679.66(1212.92, 2251.05) | 0.64(0.26, 0.94) | 0.212(0.198, 0.225) | 0.126(0.113, 0.138) | 0.207(0.199, 0.215) | 0.352(0.184, 0.520) |
| Nepal | 21964.03(20659.65, 23375.35) | 4921.41(4420.83, 5509.79) | 2261.78(1674.3, 2995.9) | 2.55(1.86, 3.4) | 0.190(0.161, 0.219) | -0.071(-0.114, -0.028) | 0.181(0.136, 0.226) | 0.808(0.488, 1.129) |
| Netherlands | 20230.82(19094.34, 21475.11) | 4759.04(4304.28, 5226.36) | 1960.75(1437.77, 2610.01) | 1.89(1.59, 2.08) | 0.016(-0.001, 0.032) | -0.025(-0.075, 0.024) | -0.027(-0.045, -0.009) | -1.147(-1.454, -0.839) |
| New Zealand | 21870.17(20613.96, 23298.89) | 6042.01(5470.47, 6696.9) | 2152.43(1589.91, 2892.83) | 2.07(1.79, 2.29) | 0.112(0.086, 0.137) | -0.080(-0.106, -0.054) | 0.074(0.047, 0.101) | -0.446(-0.818, -0.071) |
| Nicaragua | 21087.36(19856.43, 22407.75) | 4365.56(3927.82, 4834.4) | 2081.89(1530.69, 2755.57) | 1.82(1.44, 2.25) | 0.247(0.244, 0.250) | 0.046(0.038, 0.053) | 0.236(0.228, 0.245) | 0.209(0.013, 0.405) |
| Niger | 16524.88(15528.48, 17533.61) | 4130.27(3727.38, 4561.11) | 1588.55(1158.45, 2127.84) | 0.73(0.47, 1.08) | 0.125(0.115, 0.136) | 0.078(0.052, 0.105) | 0.157(0.142, 0.172) | -0.034(-0.122, 0.053) |
| Nigeria | 18129.92(17103.17, 19165.64) | 4340.86(3930.88, 4777.27) | 1733.5(1263.09, 2307.54) | 0.77(0.46, 1.08) | 0.185(0.168, 0.202) | 0.019(-0.015, 0.054) | 0.217(0.199, 0.235) | 0.667(0.604, 0.730) |
| Niue | 18751.39(17754.16, 19860.23) | 3986.14(3621.62, 4370.93) | 1713.23(1242.64, 2295.12) | 0.71(0.3, 0.97) | 0.182(0.172, 0.193) | 0.049(0.038, 0.060) | 0.148(0.138, 0.158) | 0.520(0.028, 1.015) |
| North Macedonia | 19900.37(18567.57, 21241.2) | 5865.87(5268.3, 6497.99) | 1976.37(1441.17, 2676.11) | 0.38(0.28, 0.5) | 0.167(0.159, 0.175) | 0.020(0.015, 0.025) | 0.138(0.127, 0.149) | -0.870(-1.077, -0.662) |
| Northern Mariana Islands | 18603.22(17583.52, 19728.68) | 3955.58(3590.82, 4373.03) | 1679.85(1203.88, 2267.15) | 0.35(0.28, 0.47) | 0.088(0.059, 0.116) | 0.007(-0.020, 0.033) | 0.066(0.037, 0.096) | -0.934(-1.168, -0.700) |
| Norway | 19640.32(18588.99, 20811.37) | 5012.99(4534.57, 5509.4) | 1907.41(1399.52, 2561.68) | 1.71(1.46, 1.88) | -0.002(-0.036, 0.033) | -0.166(-0.194, -0.138) | -0.123(-0.160, -0.087) | -1.685(-1.959, -1.412) |
| Oman | 19764.61(18618.86, 20953.06) | 4672.8(4241.49, 5153.58) | 1904.3(1380.98, 2520.6) | 0.75(0.55, 0.96) | 0.423(0.407, 0.440) | 0.048(0.036, 0.060) | 0.436(0.417, 0.454) | 1.059(0.766, 1.352) |
| Pakistan | 19066.96(17905.08, 20304.31) | 4261.62(3792.92, 4737.75) | 1895.55(1405.01, 2504.79) | 3.28(2.44, 4.39) | 0.455(0.413, 0.496) | 0.351(0.307, 0.396) | 0.510(0.457, 0.562) | 0.456(0.239, 0.675) |
| Palau | 18289.89(17279.25, 19422.46) | 3926.82(3592.09, 4296.83) | 1615.47(1157.74, 2180.46) | 0.14(0.11, 0.18) | 0.121(0.106, 0.136) | 0.013(-0.005, 0.031) | 0.066(0.050, 0.082) | 0.307(0.191, 0.423) |
| Palestine | 19464.4(18215.28, 20796.01) | 4856.57(4373.7, 5341.51) | 1879.74(1363.42, 2527.09) | 0.44(0.25, 0.54) | 0.174(0.153, 0.195) | -0.020(-0.025, -0.016) | 0.100(0.076, 0.125) | -0.329(-0.515, -0.142) |
| Panama | 21877.79(20630.32, 23182.6) | 4285.47(3868.19, 4713.5) | 2157.25(1587.36, 2869.49) | 1.5(1.19, 1.79) | 0.218(0.213, 0.223) | 0.065(0.054, 0.076) | 0.216(0.208, 0.224) | 0.379(0.287, 0.470) |
| Papua New Guinea | 16457.99(15473.17, 17494.05) | 3790.72(3428.63, 4177.28) | 1501.79(1089.6, 2017.28) | 0.32(0.09, 0.56) | 0.103(0.099, 0.107) | 0.027(0.013, 0.040) | 0.083(0.076, 0.091) | 0.002(-0.080, 0.084) |
| Paraguay | 21193.31(19977.94, 22497.99) | 4429.64(3994, 4902.41) | 2098.47(1551.66, 2772.33) | 2.04(1.41, 2.66) | 0.135(0.128, 0.143) | 0.091(0.051, 0.131) | 0.210(0.181, 0.238) | 2.303(2.029, 2.579) |
| Peru | 19528.13(18487.18, 20703.41) | 3723.27(3383.31, 4090.06) | 1829.33(1324.31, 2447.48) | 0.92(0.69, 1.22) | 0.263(0.254, 0.272) | 0.160(0.145, 0.176) | 0.293(0.282, 0.304) | -1.456(-1.844, -1.066) |
| Philippines | 16810.54(15857.01, 17815.29) | 3723.5(3386.05, 4091.53) | 1598.97(1169.55, 2128.56) | 1.37(1.12, 1.62) | 0.131(0.117, 0.144) | -0.016(-0.028, -0.003) | 0.095(0.081, 0.109) | 0.205(0.133, 0.278) |
| Poland | 21656.4(20283.65, 23008.56) | 6515.74(5870.6, 7224.96) | 2213.29(1629.62, 2982.94) | 0.79(0.72, 0.86) | 0.097(0.091, 0.103) | -0.037(-0.047, -0.028) | 0.002(-0.016, 0.019) | -3.201(-3.572, -2.828) |
| Portugal | 21412.06(20234.3, 22657.68) | 5374.23(4860.9, 5923.03) | 2139.25(1561.64, 2875.05) | 0.94(0.81, 1.03) | 0.241(0.201, 0.281) | -0.045(-0.066, -0.024) | 0.229(0.189, 0.268) | -0.958(-1.314, -0.601) |
| Puerto Rico | 19298.94(18142.49, 20384.92) | 3822.78(3469.84, 4180.65) | 1758.46(1270.3, 2356.17) | 1.22(1, 1.45) | 0.197(0.191, 0.203) | 0.039(0.028, 0.049) | 0.160(0.148, 0.172) | -1.389(-1.595, -1.183) |
| Qatar | 20089.15(19000.64, 21317.46) | 4706.06(4260.13, 5164.46) | 1907.34(1388.32, 2544.67) | 0.27(0.18, 0.34) | 0.243(0.221, 0.264) | 0.032(0.008, 0.055) | 0.245(0.221, 0.268) | -0.277(-0.848, 0.297) |
| Republic of Korea | 23566.8(22272.99, 24986.72) | 5068.42(4577.82, 5594.5) | 2281.76(1675.14, 3062.27) | 1.35(0.99, 1.77) | 0.294(0.250, 0.338) | -0.099(-0.118, -0.080) | 0.256(0.215, 0.297) | -2.757(-3.170, -2.343) |
| Republic of Moldova | 19427.35(18227.69, 20677.57) | 5498.88(4948.56, 6110.35) | 1922.93(1406.48, 2609.82) | 0.85(0.74, 0.97) | 0.221(0.199, 0.242) | 0.001(-0.005, 0.008) | 0.209(0.186, 0.231) | 1.173(0.340, 2.012) |
| Romania | 20770.55(19393.75, 22182.43) | 6210.39(5580.95, 6921.08) | 2116.41(1545.64, 2868.69) | 0.3(0.26, 0.34) | 0.036(0.029, 0.043) | -0.099(-0.110, -0.087) | 0.002(-0.009, 0.013) | -1.255(-1.403, -1.108) |
| Russian Federation | 20226.76(18998.89, 21484.29) | 5830.97(5259.87, 6416.93) | 2003.15(1470.18, 2717.46) | 1.83(1.67, 1.98) | 0.134(0.093, 0.175) | 0.016(-0.008, 0.039) | 0.141(0.089, 0.192) | 1.465(0.568, 2.370) |
| Rwanda | 15963.34(14567.91, 17131.99) | 4335.81(3913.67, 4773.26) | 1531.29(1095.14, 2065.12) | 0.58(0.33, 1.21) | 0.050(-0.039, 0.140) | -0.066(-0.082, -0.050) | -0.018(-0.118, 0.083) | -2.141(-2.466, -1.815) |
| Saint Kitts and Nevis | 18704.97(17627.17, 19866.94) | 3758.23(3407.14, 4112.46) | 1759.25(1291.8, 2333.49) | 3.71(3.15, 4.28) | 0.172(0.167, 0.176) | -0.004(-0.006, -0.002) | 0.076(0.055, 0.097) | -0.429(-0.740, -0.116) |
| Saint Lucia | 18517.66(17423.76, 19550.3) | 3796.99(3432.23, 4152.5) | 1731.01(1260.52, 2288.51) | 2.63(2.19, 3.12) | 0.179(0.167, 0.191) | -0.038(-0.044, -0.033) | 0.100(0.089, 0.110) | -1.294(-1.693, -0.893) |
| Saint Vincent and the Grenadines | 18090.95(17003.24, 19168.26) | 3713.51(3351.05, 4068.88) | 1680.57(1226.94, 2233.52) | 2.36(2.04, 2.68) | 0.201(0.196, 0.205) | 0.025(0.021, 0.029) | 0.180(0.166, 0.194) | 0.850(0.488, 1.212) |
| Samoa | 18317.27(17327.05, 19431.2) | 3998.58(3628, 4379.06) | 1662.12(1197.69, 2222.21) | 0.44(0.17, 0.73) | 0.068(0.053, 0.082) | -0.082(-0.102, -0.062) | -0.015(-0.036, 0.005) | 0.299(0.207, 0.390) |
| San Marino | 20900.03(19634.56, 22168.25) | 5093.74(4602.59, 5607.84) | 2033.83(1476.03, 2736.7) | 0.36(0.23, 0.51) | 0.042(0.035, 0.049) | -0.054(-0.062, -0.046) | -0.001(-0.009, 0.006) | -1.325(-1.650, -0.999) |
| Sao Tome and Principe | 17363.56(16308.64, 18385.34) | 3883.84(3529.58, 4268.76) | 1595.26(1157.58, 2162.75) | 0.87(0.6, 1.11) | 0.189(0.181, 0.197) | -0.014(-0.032, 0.003) | 0.114(0.102, 0.126) | 0.490(0.329, 0.652) |
| Saudi Arabia | 19578.92(18368.74, 20846.15) | 4793.72(4327.97, 5276.3) | 1865.68(1364.42, 2483.32) | 0.92(0.65, 1.2) | 0.269(0.232, 0.306) | 0.107(0.096, 0.118) | 0.302(0.277, 0.328) | 1.632(1.350, 1.915) |
| Senegal | 16995.15(15946.16, 18054.77) | 3918.05(3535.14, 4307.91) | 1586.82(1147.6, 2112.51) | 0.87(0.6, 1.16) | 0.075(0.061, 0.089) | -0.098(-0.121, -0.075) | 0.042(0.026, 0.058) | 0.456(0.378, 0.533) |
| Serbia | 20987.92(19580.33, 22360.35) | 6199.99(5579.77, 6868.74) | 2145.96(1562.35, 2897.4) | 0.84(0.66, 1.08) | 0.178(0.172, 0.185) | 0.024(0.020, 0.027) | 0.148(0.139, 0.157) | -1.106(-1.258, -0.953) |
| Seychelles | 17686.33(16700.65, 18803.93) | 3411.96(3116.43, 3748.19) | 1585.23(1144.38, 2129.28) | 0.16(0.11, 0.19) | 0.173(0.155, 0.191) | -0.052(-0.060, -0.045) | 0.087(0.071, 0.104) | 0.118(-0.035, 0.272) |
| Sierra Leone | 16841.43(15808.6, 17892.33) | 4059.15(3669.41, 4475.34) | 1593.44(1149.45, 2127.34) | 0.75(0.44, 1.08) | 0.043(0.028, 0.059) | -0.100(-0.121, -0.080) | 0.011(-0.003, 0.026) | 0.825(0.662, 0.989) |
| Singapore | 22665.71(21389.27, 24028.36) | 4532.4(4105.22, 4966.14) | 2148.61(1549.24, 2888.58) | 0.63(0.55, 0.7) | 0.184(0.164, 0.203) | -0.067(-0.106, -0.028) | 0.162(0.132, 0.192) | -3.232(-3.441, -3.022) |
| Slovakia | 20990.22(19677.19, 22386.68) | 6162.57(5556.76, 6839.54) | 2106.31(1547.45, 2846.31) | 0.44(0.33, 0.55) | 0.060(0.049, 0.071) | -0.051(-0.056, -0.046) | 0.035(0.019, 0.050) | -0.980(-1.092, -0.868) |
| Slovenia | 20117.46(18762.11, 21554.38) | 5911.06(5320.91, 6545.19) | 2008.85(1463.12, 2708.98) | 1.01(0.84, 1.18) | 0.089(0.074, 0.103) | -0.025(-0.037, -0.013) | 0.028(0.001, 0.055) | -2.222(-2.541, -1.901) |
| Solomon Islands | 17191.14(16211.95, 18229.57) | 3988.73(3635.43, 4406.32) | 1567.49(1136.82, 2110.52) | 0.42(0.15, 0.68) | 0.257(0.252, 0.263) | 0.213(0.198, 0.228) | 0.275(0.264, 0.286) | 0.706(0.615, 0.798) |
| Somalia | 15798.73(14790.77, 16869.36) | 4185.3(3772.5, 4629.08) | 1512.25(1091.3, 2033.62) | 0.68(0.35, 1.47) | 0.029(0.024, 0.033) | 0.003(-0.009, 0.016) | 0.024(0.015, 0.033) | -0.178(-0.271, -0.085) |
| South Africa | 18345.3(17324.11, 19365.55) | 4115.94(3741.54, 4507.8) | 1712.76(1261.92, 2267.3) | 2.22(1.89, 2.58) | 0.147(0.138, 0.157) | -0.091(-0.101, -0.081) | 0.049(0.036, 0.063) | -0.164(-0.497, 0.169) |
| South Sudan | 15970.41(14964.48, 16963.62) | 4106.87(3716, 4506.53) | 1502.42(1098.02, 2006.24) | 0.66(0.37, 1.2) | 0.049(0.046, 0.053) | -0.001(-0.012, 0.010) | 0.032(0.027, 0.037) | 0.266(0.034, 0.499) |
| Spain | 18910.74(17859.12, 20087.81) | 4586.89(4103.11, 5079.55) | 1776.89(1279.09, 2383.39) | 1.26(1.06, 1.4) | -0.042(-0.144, 0.061) | -0.148(-0.269, -0.027) | -0.248(-0.425, -0.071) | -4.021(-5.044, -2.987) |
| Sri Lanka | 16910.8(15957.67, 18010.32) | 3469.27(3143.64, 3810.22) | 1531.42(1108.91, 2060.62) | 0.18(0.11, 0.25) | 0.242(0.228, 0.257) | 0.041(0.030, 0.052) | 0.192(0.180, 0.204) | 0.765(0.426, 1.106) |
| Sudan | 19617.7(18391.35, 20821.77) | 4869.69(4398.04, 5339.12) | 1947.39(1410.77, 2586.45) | 0.77(0.5, 1.17) | 0.304(0.284, 0.323) | 0.057(0.055, 0.059) | 0.287(0.270, 0.304) | 1.251(1.141, 1.362) |
| Suriname | 18600.7(17523.05, 19712.99) | 3816.77(3457.62, 4195.71) | 1714.16(1256.1, 2279.32) | 1.91(1.46, 2.44) | 0.214(0.207, 0.222) | 0.093(0.083, 0.103) | 0.191(0.179, 0.203) | 0.142(-0.032, 0.316) |
| Sweden | 18812.6(17657.14, 20066.89) | 4317.04(3866.88, 4780.12) | 1821.59(1312.36, 2449.19) | 1.31(1.11, 1.48) | 0.346(0.251, 0.442) | 0.473(0.332, 0.614) | 0.390(0.288, 0.491) | -0.789(-1.140, -0.436) |
| Switzerland | 21252.64(20036.22, 22642.86) | 5315.36(4783.03, 5870.99) | 2114.65(1538.55, 2822.6) | 1.24(0.99, 1.4) | 0.081(0.021, 0.142) | -0.095(-0.166, -0.025) | 0.083(0.001, 0.166) | -1.332(-1.515, -1.149) |
| Syrian Arab Republic | 19686.98(18294.61, 21008.78) | 4967.69(4475.57, 5478.67) | 1907.57(1372.91, 2583.16) | 0.25(0.17, 0.33) | 0.138(0.102, 0.173) | 0.044(0.029, 0.059) | 0.072(0.025, 0.119) | 0.630(0.377, 0.884) |
| Taiwan (Province of China) | 20859.24(20046.27, 21739.04) | 4622.49(4327.21, 4917.92) | 2052.1(1520.02, 2728.89) | 1.45(1.29, 1.63) | 0.432(0.385, 0.478) | 0.319(0.258, 0.379) | 0.575(0.517, 0.632) | 1.833(1.452, 2.215) |
| Tajikistan | 16936.9(15882.67, 18042.42) | 4738.63(4250.99, 5257) | 1633.91(1191.15, 2227.65) | 0.21(0.09, 0.33) | 0.128(0.101, 0.154) | -0.044(-0.048, -0.039) | 0.101(0.077, 0.125) | 4.555(3.827, 5.287) |
| Thailand | 18109.67(17131.39, 19211.72) | 3341.86(3025.65, 3671.99) | 1729.08(1271.21, 2294.82) | 1.97(1.44, 2.57) | 0.246(0.221, 0.272) | 0.209(0.163, 0.254) | 0.187(0.167, 0.208) | -0.455(-0.698, -0.210) |
| Timor-Leste | 15706.01(14659.92, 16776.33) | 3325.12(3007.68, 3666.35) | 1461.54(1058.78, 1962.13) | 1.12(0.48, 1.9) | 0.200(0.175, 0.225) | -0.052(-0.066, -0.037) | 0.187(0.142, 0.233) | 0.994(0.729, 1.261) |
| Togo | 17343.48(16327.14, 18379.39) | 4110.75(3735.84, 4527.59) | 1650.36(1194, 2199.07) | 0.85(0.52, 1.19) | 0.067(0.056, 0.077) | -0.110(-0.146, -0.074) | 0.032(0.013, 0.051) | 0.921(0.852, 0.990) |
| Tokelau | 18377.18(17316.31, 19421.57) | 3957.77(3594.76, 4347.51) | 1683.06(1225.87, 2239.3) | 0.67(0.26, 0.95) | 0.231(0.222, 0.241) | 0.054(0.042, 0.067) | 0.184(0.175, 0.193) | 0.139(-0.401, 0.681) |
| Tonga | 18506.49(17476.52, 19566.12) | 4014.31(3648.32, 4388.01) | 1743.81(1269.14, 2331.74) | 1.76(1.31, 2.3) | 0.114(0.094, 0.135) | -0.021(-0.038, -0.004) | 0.061(0.038, 0.085) | 0.019(-0.169, 0.207) |
| Trinidad and Tobago | 18946.75(17836.2, 20057.72) | 3810.67(3461.23, 4181.99) | 1784.89(1312.71, 2372.07) | 2.6(1.97, 3.29) | 0.188(0.183, 0.193) | 0.038(0.031, 0.044) | 0.146(0.136, 0.157) | -0.872(-1.091, -0.652) |
| Tunisia | 20633.76(19458.49, 21903.75) | 4937.04(4460.41, 5448.28) | 2031.68(1478.29, 2729.81) | 0.66(0.37, 1.2) | 0.316(0.311, 0.321) | 0.090(0.080, 0.100) | 0.317(0.309, 0.326) | 2.200(2.043, 2.357) |
| Turkey | 21160.26(19950.74, 22408.01) | 5055.69(4536.71, 5560) | 2123.48(1538.64, 2842.43) | 1.21(0.95, 1.56) | 0.289(0.249, 0.329) | 0.053(0.009, 0.098) | 0.225(0.175, 0.276) | -1.314(-1.599, -1.029) |
| Turkmenistan | 18210.45(16993.55, 19376.39) | 4903.51(4428.89, 5412.7) | 1744.25(1259.83, 2372.75) | 0.3(0.24, 0.36) | 0.210(0.191, 0.229) | 0.007(-0.000, 0.014) | 0.170(0.153, 0.187) | 11.032(9.207, 12.887) |
| Tuvalu | 18069.16(17070.48, 19128.7) | 3962.5(3600.79, 4367.65) | 1645.5(1188.72, 2211.14) | 0.41(0.17, 0.63) | 0.180(0.168, 0.191) | 0.011(-0.003, 0.025) | 0.142(0.128, 0.155) | -0.604(-0.667, -0.541) |
| Uganda | 16746.3(15686.07, 17786.72) | 4227.37(3802.08, 4679.83) | 1603.29(1165.91, 2155.21) | 0.54(0.29, 1.1) | 0.139(0.133, 0.145) | -0.042(-0.054, -0.031) | 0.146(0.142, 0.150) | 0.033(-0.198, 0.264) |
| Ukraine | 20918.32(19595.01, 22240.28) | 6260.6(5616.78, 6895.16) | 2109.06(1556.9, 2852.45) | 0.58(0.41, 0.77) | 0.085(0.067, 0.102) | -0.023(-0.038, -0.007) | 0.078(0.050, 0.106) | -0.090(-0.424, 0.245) |
| United Arab Emirates | 19188.66(18140.33, 20337.07) | 4472.26(4056.28, 4885.39) | 1812.95(1309.85, 2432.84) | 0.89(0.67, 1.16) | 0.280(0.268, 0.291) | 0.086(0.059, 0.112) | 0.281(0.267, 0.295) | 1.714(1.004, 2.429) |
| United Kingdom | 21889.66(20687.7, 23209.12) | 5408.66(4892.6, 5953.58) | 2194.8(1605.07, 2943.17) | 2.04(1.81, 2.16) | 0.103(0.078, 0.127) | 0.145(0.103, 0.188) | 0.118(0.090, 0.147) | -0.728(-1.101, -0.353) |
| United Republic of Tanzania | 17021.22(16005.78, 18028.04) | 4193.09(3782.47, 4621.98) | 1616.61(1177.32, 2174.4) | 0.54(0.3, 1.12) | 0.060(0.052, 0.068) | -0.043(-0.049, -0.037) | 0.079(0.076, 0.083) | -0.517(-0.564, -0.470) |
| United States of America | 27919.91(26947.1, 28824.75) | 5886.47(5472.58, 6325.24) | 2791.85(2052.27, 3646.56) | 1.51(1.35, 1.61) | 0.329(0.311, 0.346) | -0.004(-0.066, 0.058) | 0.277(0.263, 0.290) | -1.182(-1.688, -0.674) |
| United States Virgin Islands | 19273.76(18190.62, 20355.56) | 3819.95(3457.44, 4182.71) | 1765.19(1283.83, 2349.81) | 1.6(1.2, 2.13) | 0.203(0.192, 0.213) | 0.039(0.023, 0.054) | 0.166(0.151, 0.180) | -0.630(-0.851, -0.410) |
| Uruguay | 24920.08(23605.15, 26433.25) | 5333.7(4801.28, 5894.13) | 2543.59(1879.39, 3398.69) | 1.7(1.55, 1.84) | 0.316(0.289, 0.343) | 0.174(0.135, 0.212) | 0.365(0.328, 0.402) | 0.529(0.352, 0.707) |
| Uzbekistan | 18402.86(17264.52, 19530.64) | 4999.98(4525.13, 5532.89) | 1791.12(1291.51, 2432.44) | 0.71(0.59, 0.85) | 0.205(0.184, 0.225) | 0.045(0.034, 0.055) | 0.227(0.197, 0.257) | 6.879(5.427, 8.351) |
| Vanuatu | 17602.94(16631.24, 18650.11) | 4061.48(3668.13, 4484.67) | 1651.68(1206.52, 2212.07) | 0.42(0.15, 0.7) | 0.170(0.160, 0.180) | 0.071(0.052, 0.091) | 0.165(0.147, 0.182) | 0.571(0.476, 0.666) |
| Venezuela (Bolivarian Republic of) | 21156.13(20008.67, 22371.65) | 4153.12(3748.85, 4547.29) | 2102.65(1541.99, 2787.12) | 2.61(1.97, 3.4) | 0.094(0.080, 0.108) | -0.051(-0.060, -0.041) | 0.098(0.080, 0.116) | 0.030(-0.213, 0.273) |
| Viet Nam | 16408.01(15369.21, 17507.53) | 3508.15(3181.87, 3862.37) | 1555.26(1137.54, 2084.67) | 0.93(0.46, 1.24) | 0.265(0.252, 0.278) | 0.048(0.027, 0.069) | 0.240(0.220, 0.261) | 0.464(0.397, 0.532) |
| Yemen | 18923.64(17771.68, 20140.18) | 4826.16(4338.59, 5305.04) | 1861.32(1350.54, 2457.22) | 0.59(0.3, 0.9) | 0.215(0.202, 0.229) | -0.030(-0.037, -0.023) | 0.177(0.162, 0.192) | 1.325(1.181, 1.469) |
| Zambia | 16673.77(15706.08, 17678.89) | 4006.61(3629.38, 4387.15) | 1554.89(1138.38, 2062.24) | 0.61(0.36, 0.93) | 0.181(0.158, 0.203) | 0.091(0.053, 0.129) | 0.195(0.169, 0.221) | -0.903(-1.075, -0.731) |
| Zimbabwe | 17277.33(16264.25, 18303.11) | 4175.39(3750.22, 4621.36) | 1672.54(1227.87, 2212.01) | 1.42(0.67, 1.93) | 0.127(0.116, 0.139) | 0.125(0.120, 0.129) | 0.200(0.189, 0.210) | 1.382(0.928, 1.838) |

EAPC = estimated annual percentage change; SDI = socio-demographic index; 95% UI = 95% uncertainty interval; 95% CI = 95% confidence interval.
