# Supplementary figures and images for: Global, regional, and national burden of musculoskeletal disorders, 1990–2021: an analysis of the global burden of disease study 2021 and forecast to 2035
Source: Front Public Health. 2025 Aug 1;13:1562701. doi: 10.3389/fpubh.2025.1562701 (PMC12354483; doi:10.3389/fpubh.2025.1562701)

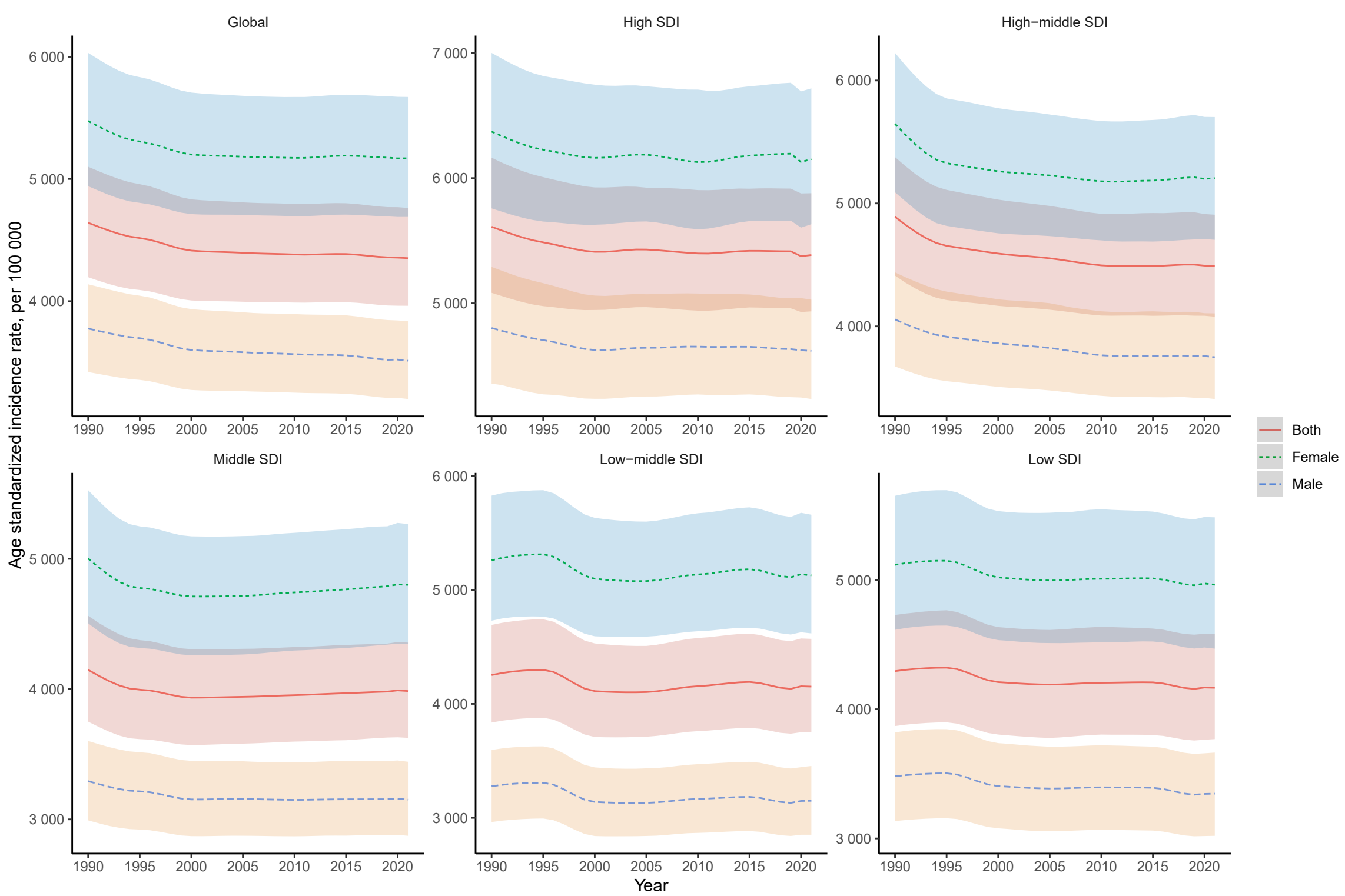

Supplement: Supplementary file 11 [file Data_Sheet_1.pdf]

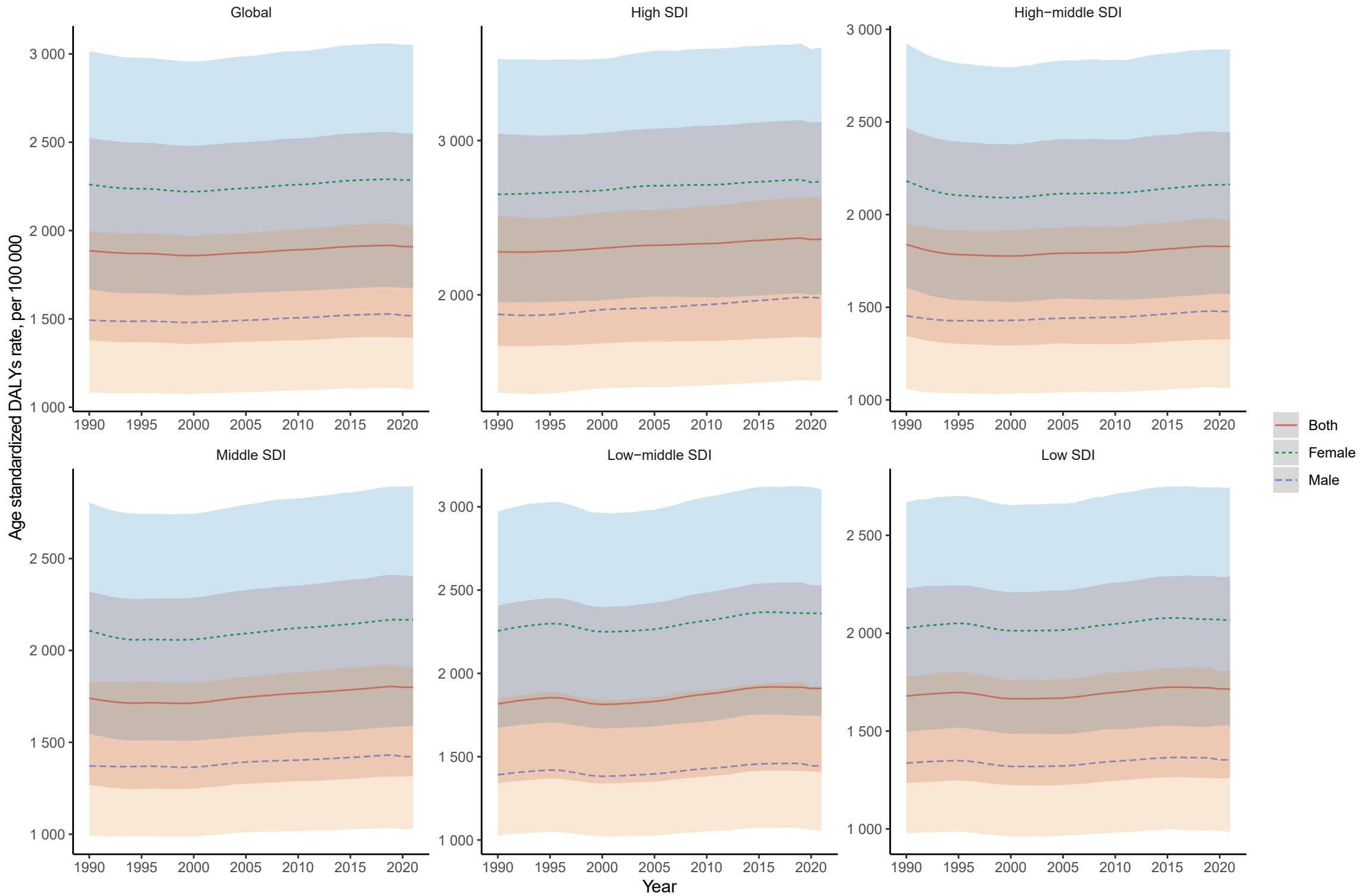

Supplement: Supplementary file 12 [file Data_Sheet_2.pdf]

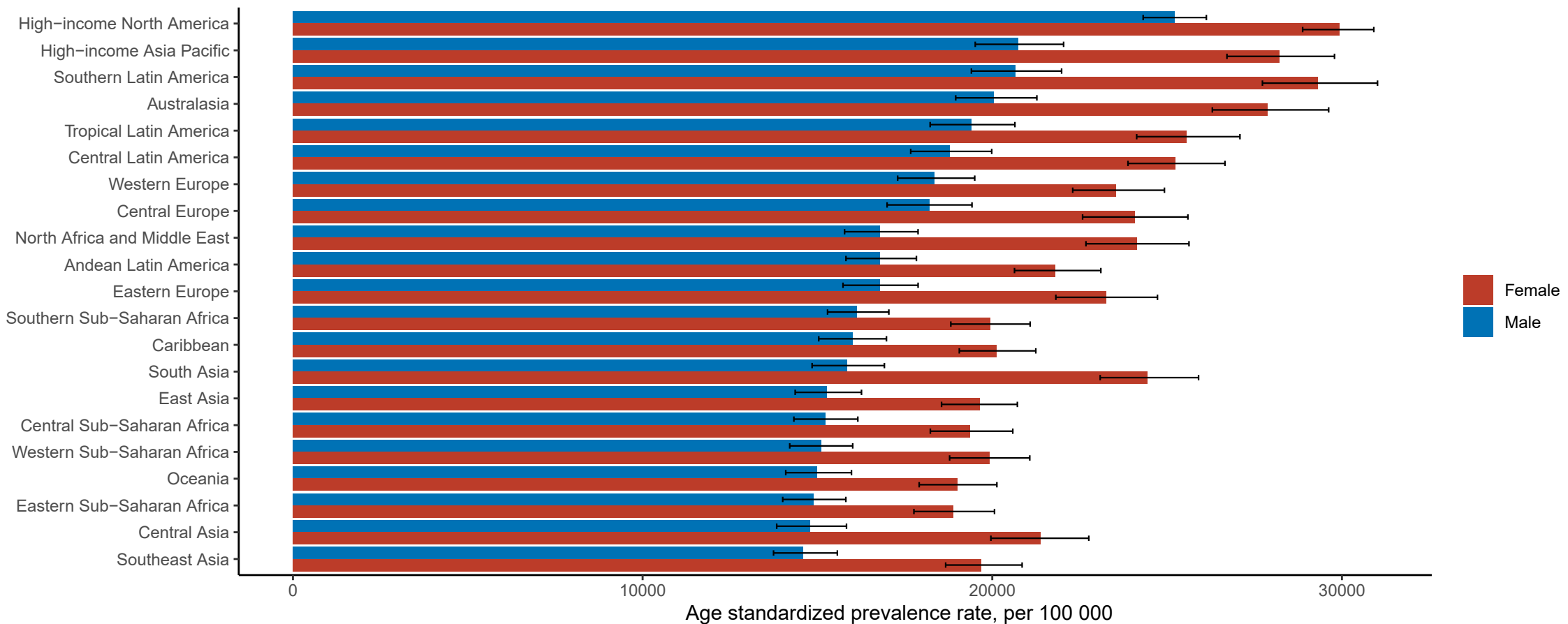

Supplement: Supplementary file 14 [file Data_Sheet_4.pdf]

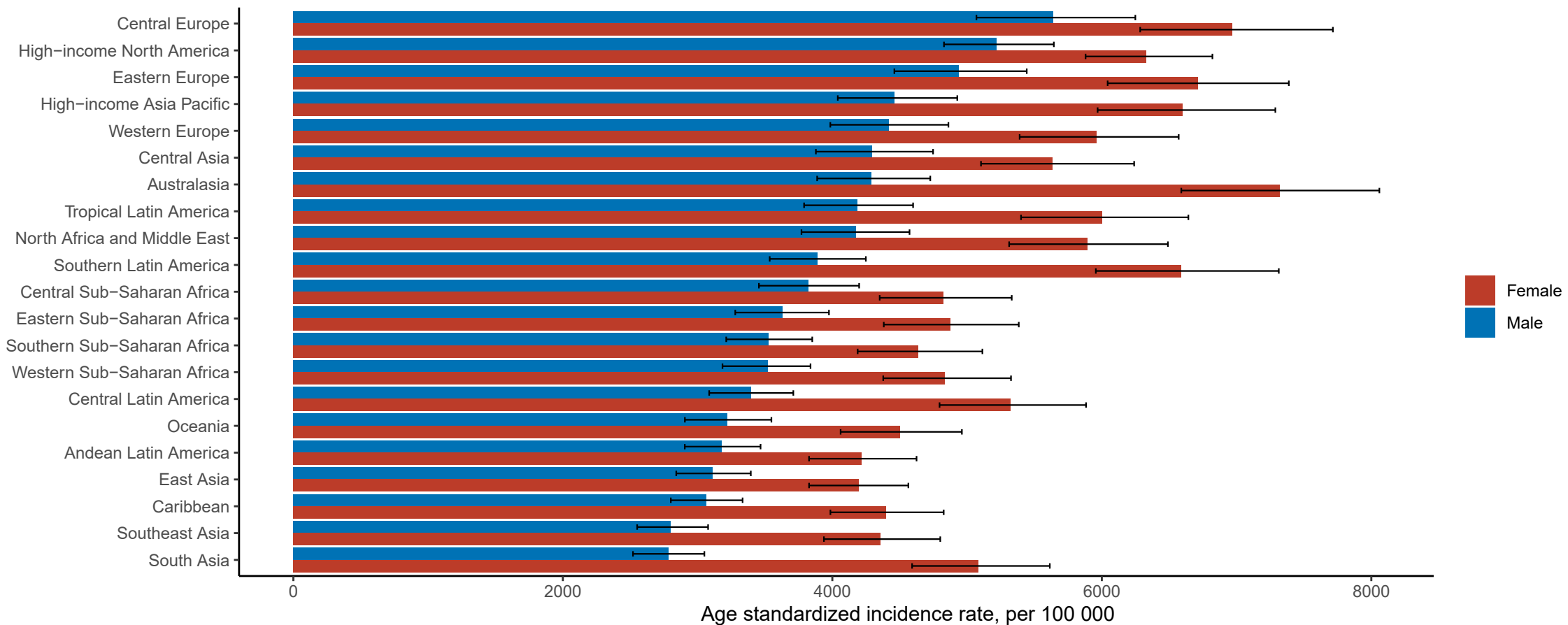

Supplement: Supplementary file 15 [file Data_Sheet_5.pdf]

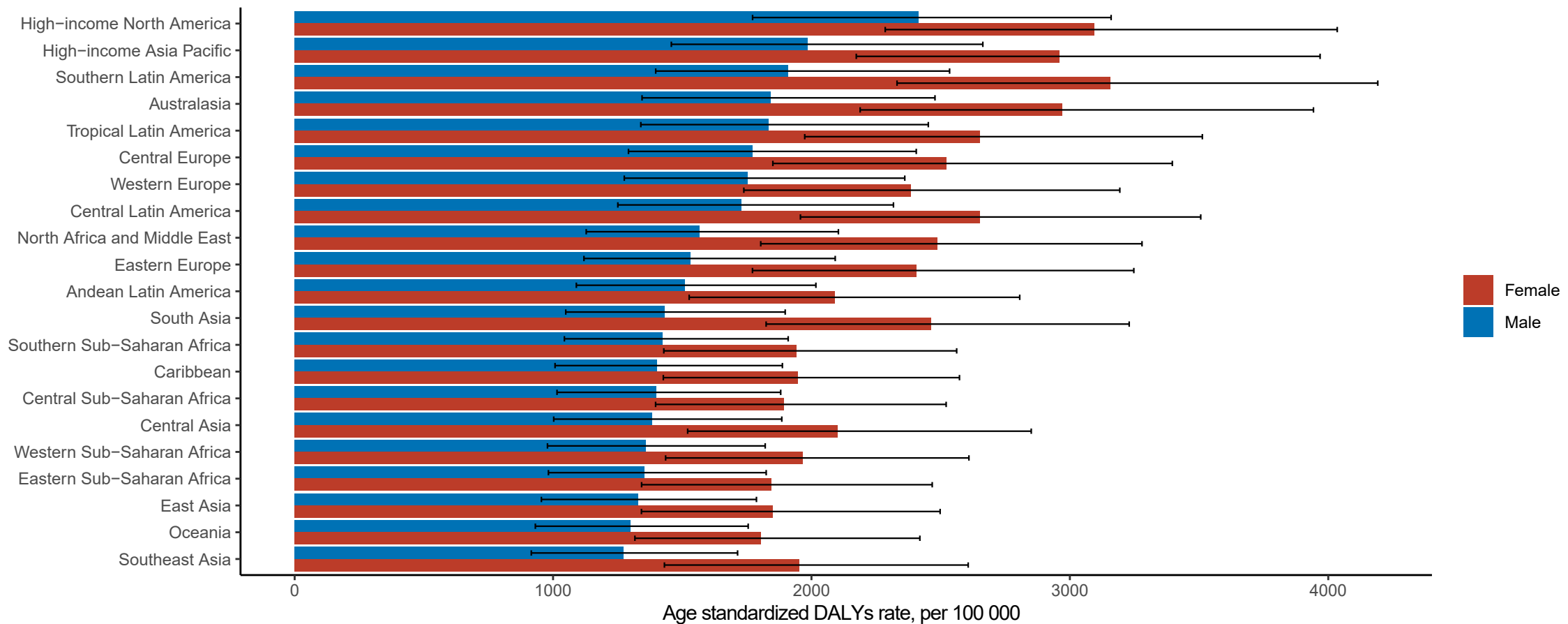

Supplement: Supplementary file 16 [file Data_Sheet_6.pdf]

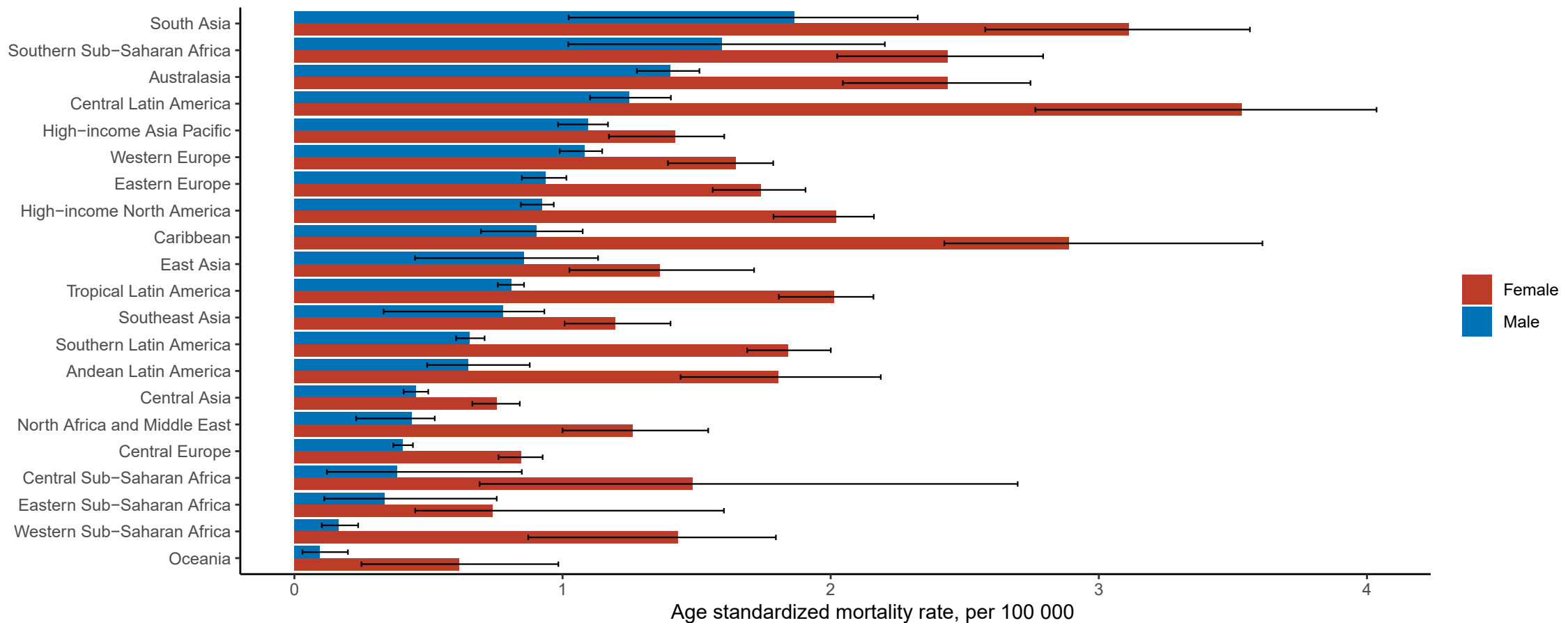

Supplement: Supplementary file 17 [file Data_Sheet_7.pdf]

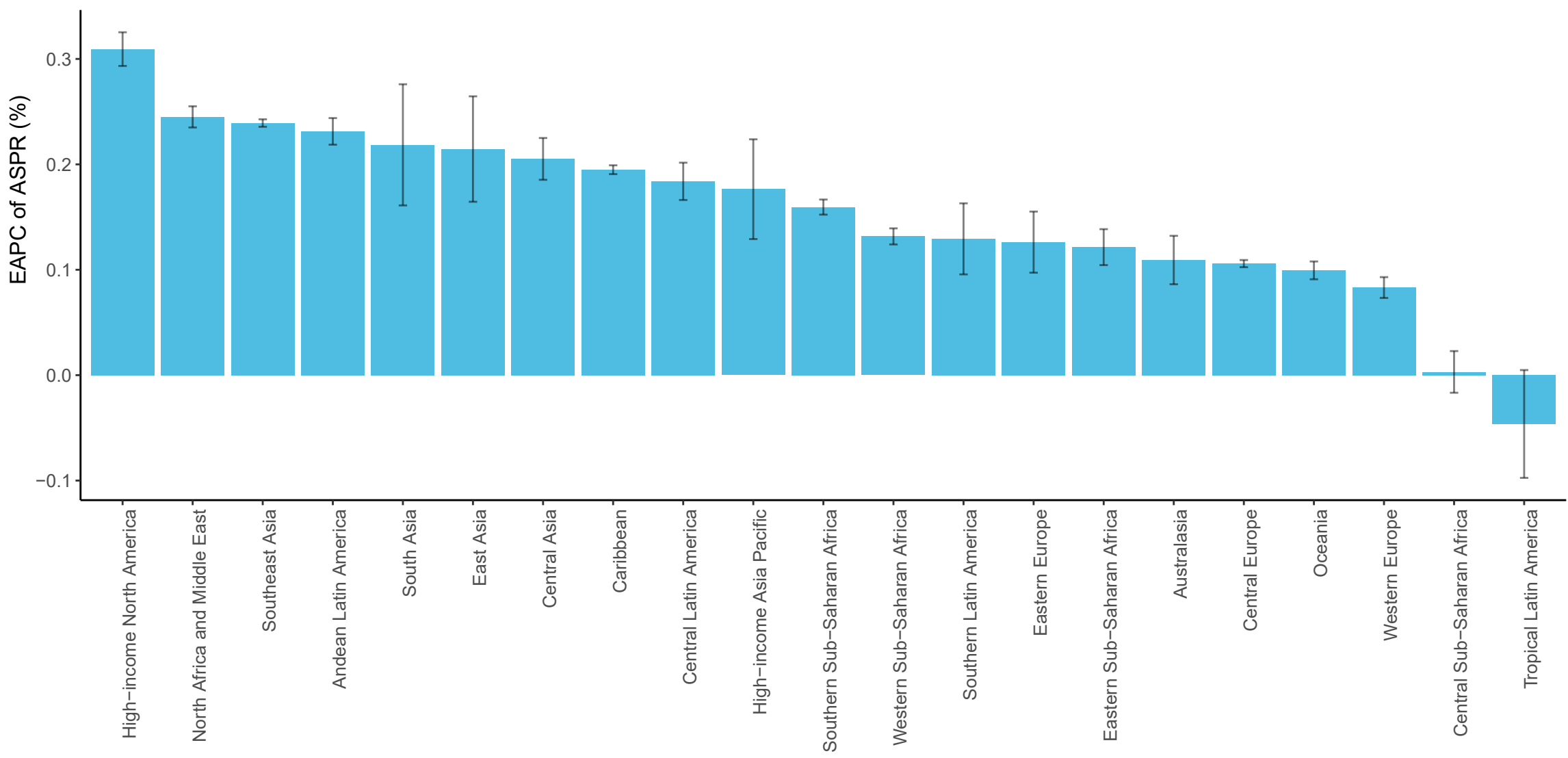

Supplement: Supplementary file 18 [file Data_Sheet_8.pdf]

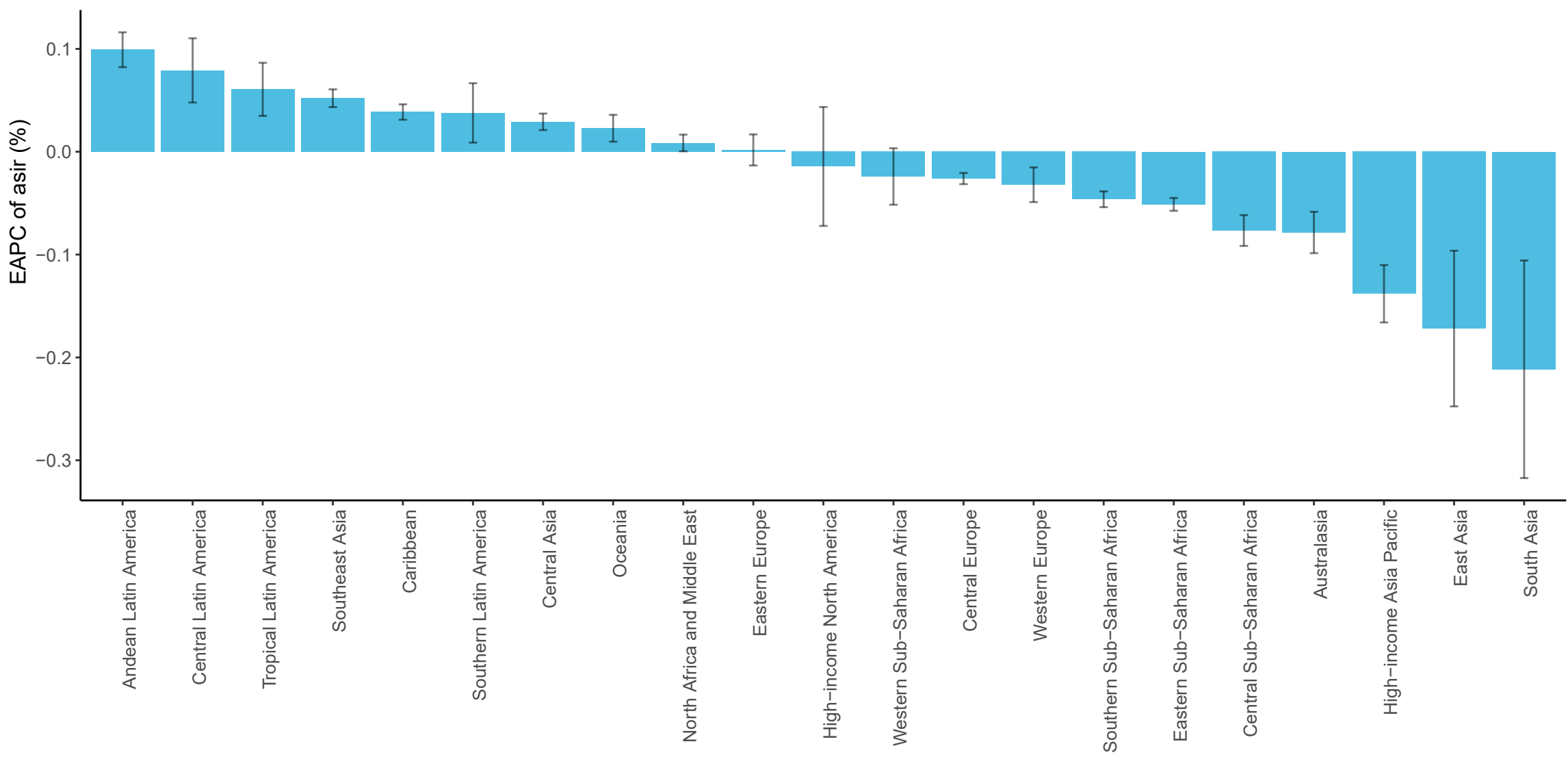

Supplement: Supplementary file 19 [file Data_Sheet_9.pdf]

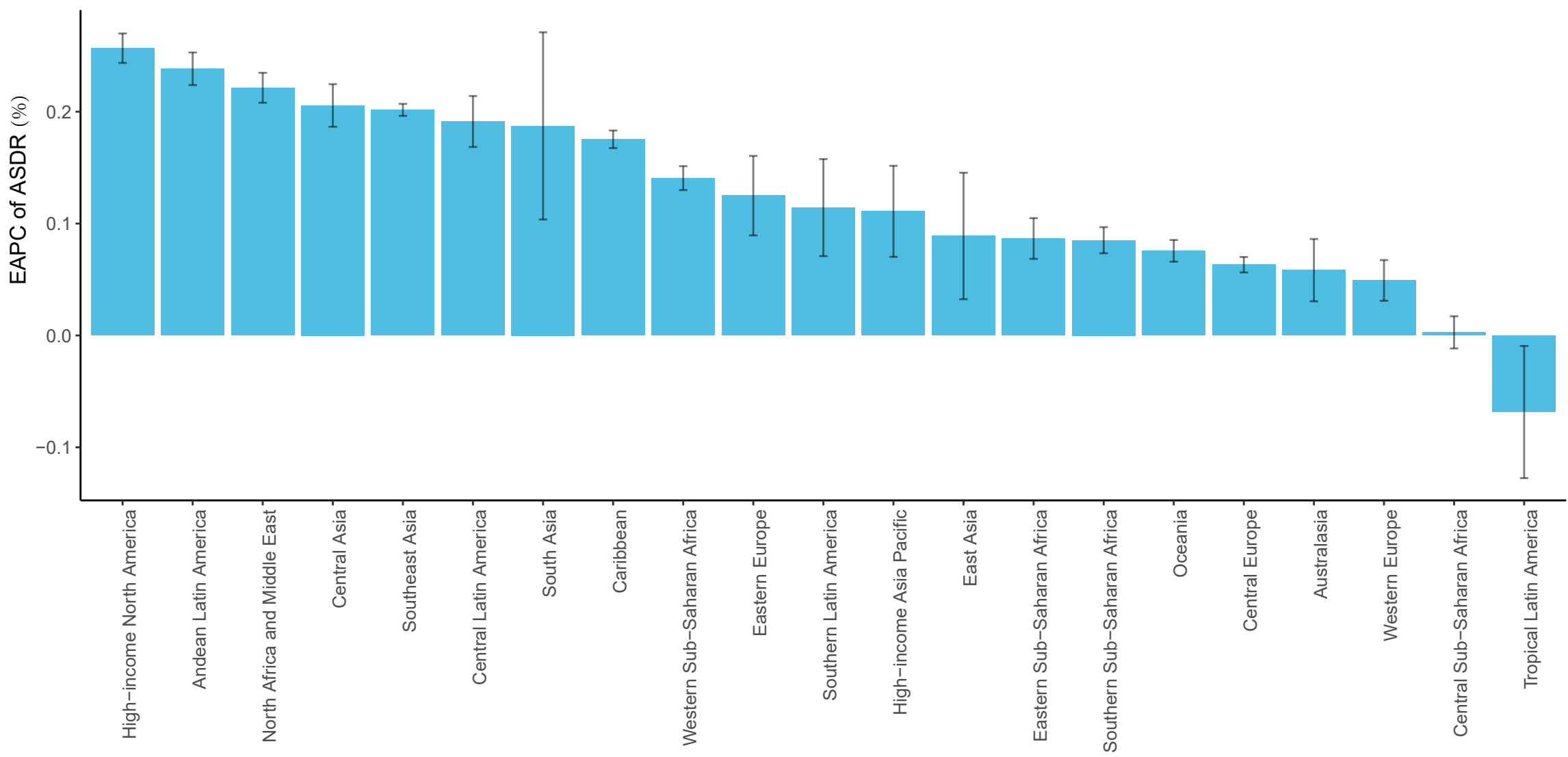

Supplement: Supplementary file 20 [file Data_Sheet_10.pdf]

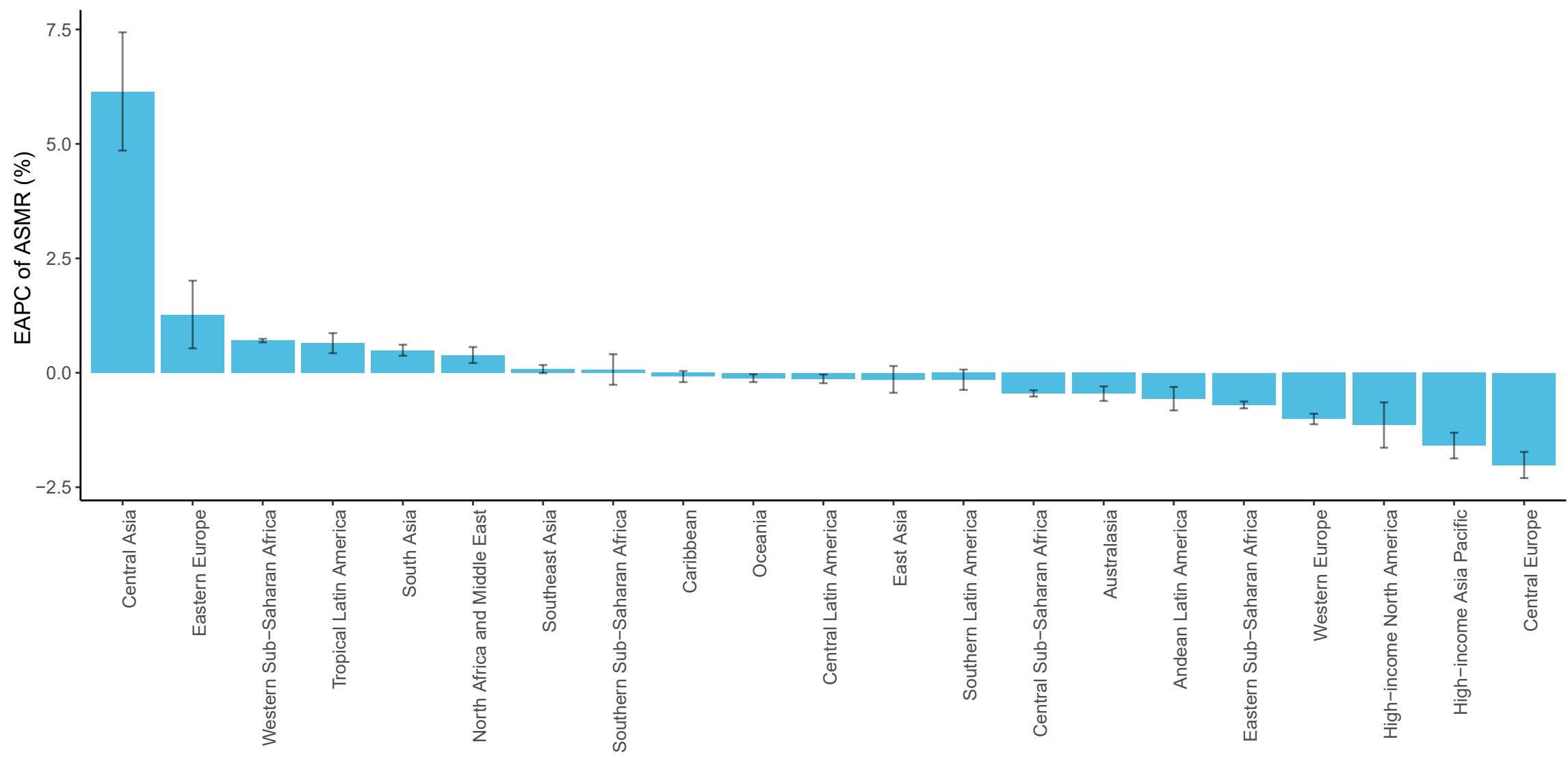

Supplement: Supplementary file 21 [file Data_Sheet_11.pdf]
