# Supplementary material for: Global, regional, and national burden of musculoskeletal disorders, 1990–2021: an analysis of the global burden of disease study 2021 and forecast to 2035
Source: Front Public Health. 2025 Aug 1;13:1562701. doi: 10.3389/fpubh.2025.1562701 (PMC12354483; doi:10.3389/fpubh.2025.1562701)
Supplement: Supplementary file 13 [file Data_Sheet_3.pdf]

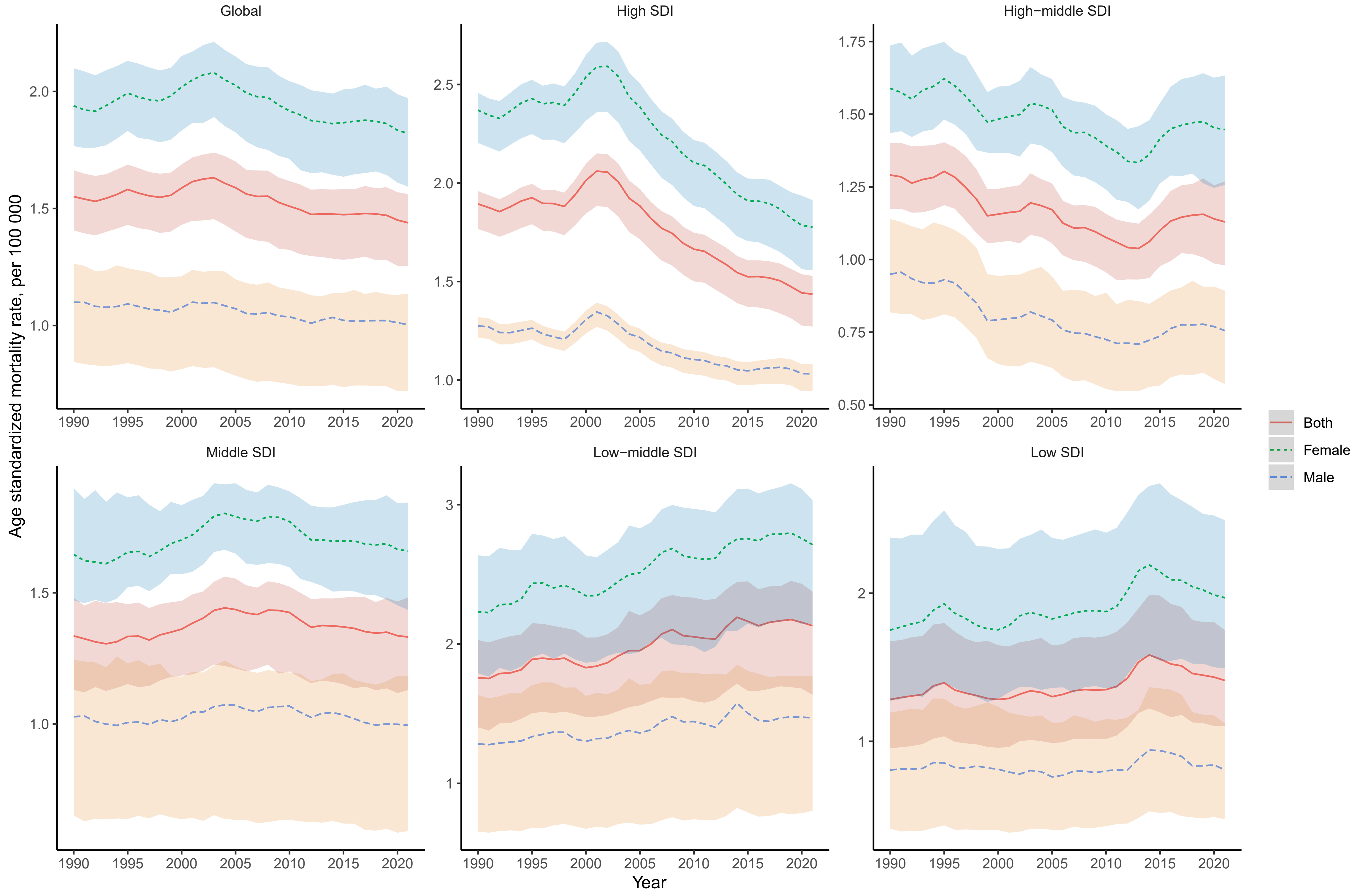

Global
High SDI
High−middle SDI
Middle SDI
Low−middle SDI
Low SDI
Age standardized mortality rate, per 100 000
Year
Both
Female
Male
